# Metabolic regulation of tumor-associated macrophage heterogeneity: insights into the tumor microenvironment and immunotherapeutic opportunities

**DOI:** 10.1186/s40364-023-00549-7

**Published:** 2024-01-07

**Authors:** Yujing Qian, Yujia Yin, Xiaocui Zheng, Zhaoyuan Liu, Xipeng Wang

**Affiliations:** 1 Department of Obstetrics and Gynecology, Xinhua Hospital Affiliated to Shanghai Jiao Tong University School of Medicine, Shanghai, 200092 China; 2 Department of Immunology and Microbiology, Shanghai Institute of Immunology, Shanghai Jiao Tong University School of Medicine, Shanghai, 200025 China

**Keywords:** Tumor-associated macrophages, Tumor microenvironment, Metabolic reprogramming, Single-cell omics, Immunotherapy

## Abstract

Tumor-associated macrophages (TAMs) are a heterogeneous population that play diverse functions in tumors. Their identity is determined not only by intrinsic factors, such as origins and transcription factors, but also by external signals from the tumor microenvironment (TME), such as inflammatory signals and metabolic reprogramming. Metabolic reprogramming has rendered TAM to exhibit a spectrum of activities ranging from pro-tumorigenic to anti-tumorigenic, closely associated with tumor progression and clinical prognosis. This review implicates the diversity of TAM phenotypes and functions, how this heterogeneity has been re-evaluated with the advent of single-cell technologies, and the impact of TME metabolic reprogramming on TAMs. We also review current therapies targeting TAM metabolism and offer new insights for TAM-dependent anti-tumor immunotherapy by focusing on the critical role of different metabolic programs in TAMs.

## Highlights

1. Single-cell sequencing has advanced the phenotypic and functional diversity in TAMs compared with the traditional M1/M2 polarization paradigm.

2. The heterogeneity in origins, phenotypes, and functions of TAMs dictates their diverse roles in tumor progression.

3. The metabolic reprogramming of TAMs triggered by stimuli in the TME constitutes one of the driving factors influencing their diversity.

4. Targeting specific metabolic subsets of TAMs leads to novel tumor immunotherapeutic strategies and improves patient outcomes.

## Introduction

The tumor microenvironment (TME), an intricate assembly of nonmalignant cellular entities, vascular structures, and extracellular components, plays a cardinal role in facilitating tumor progression, metastasis, and invasion [1, 2]. Its immunological profile, marked by a high prevalence of tumor-associated macrophages (TAMs), has been associated with the prognosis of patients, cementing the integral role of the TAMs in the tumor milieu [3–6]. With TAMs playing diverse roles in tumor biology, understanding their composition can illuminate the complex dynamics of tumor progression, enhance predictive accuracy regarding disease outcomes, and inform the development of innovative anti-cancer strategies.

Conventionally, TAMs have been classified into two categories based on their activation status: pro-inflammatory M1 and anti-inflammatory M2. M1 TAMs possess tumor-killing properties, whereas M2 macrophages exhibit anti-inflammatory properties, which can indirectly promote tumor growth [7, 8]. Despite the initial appeal of this binary classification, it's increasingly acknowledged that it fails to accurately capture the diverse spectrum of macrophage profiles in vivo, especially within complex TMEs [9, 10]. Consequently, there is an increasing interest in understanding the phenotypic and functional heterogeneity of TAMs, which has been further fueled by advancements in single-cell technologies like single-cell RNA sequencing (scRNA-seq). Such revolutionary technologies have elucidated the heterogeneity of TAMs, which is intricately modulated by a myriad of both intrinsic and extrinsic factors, spanning a broad spectrum of either pro-tumorigenic or anti-tumorigenic activities. Recently, our research team undertook a comprehensive 10X Genomics single-cell transcriptomic study on 39 samples derived from primary lesions, omental metastatic sites, ascites, peripheral blood, and pelvic lymph nodes in 14 patients diagnosed with epithelial ovarian cancer. Our comparative analysis of macrophages' gene expression profiles in tumors and ascites revealed that the TME, together with cellular developmental lineage, synergistically influences the functional phenotype of these macrophages [11]. Intrinsic determinants, notably ontogeny, have been validated by the heterogeneous phenotypes and functions exhibited by TAMs of embryonic and blood monocyte origins [12]. Furthermore, distinctive features of the local microenvironment—including inflammation status, hypoxia, acidity, hyperkalemia, and nutrient deprivation, among other TME attributes—profoundly mold the TAM profile, influencing its intrinsic diversity [12]. Within these TME factors, metabolic reprogramming significantly contributes to the phenotypic and functional diversity of TAMs. The discrepant metabolites and intricately regulated metabolic pathways regulate the cellular crosstalk between TAMs and other cells in the TME. Concurrently, a plethora of research underscores that targeting TAMs' specific metabolic processes can alter their phenotype, function, and anti-tumor responses thereby enhancing cancer treatment.

Here, we focus on the heterogeneity of TAMs in light of single-cell omics and the macrophage metabolism modulation by the TME. We emphasize the altered and intricately regulated metabolic pathways that contribute to macrophage function and regulation in the TME. Given the pivotal role of metabolically distinct TAMs in tumor progression, we also highlight the necessity of targeting these specific metabolic TAM subpopulations for the development of novel immunotherapeutic strategies against cancer.

## TAMs in the era of single-cell omics: The heterogeneity of origins, phenotypes and functions

**Fig. 1:**
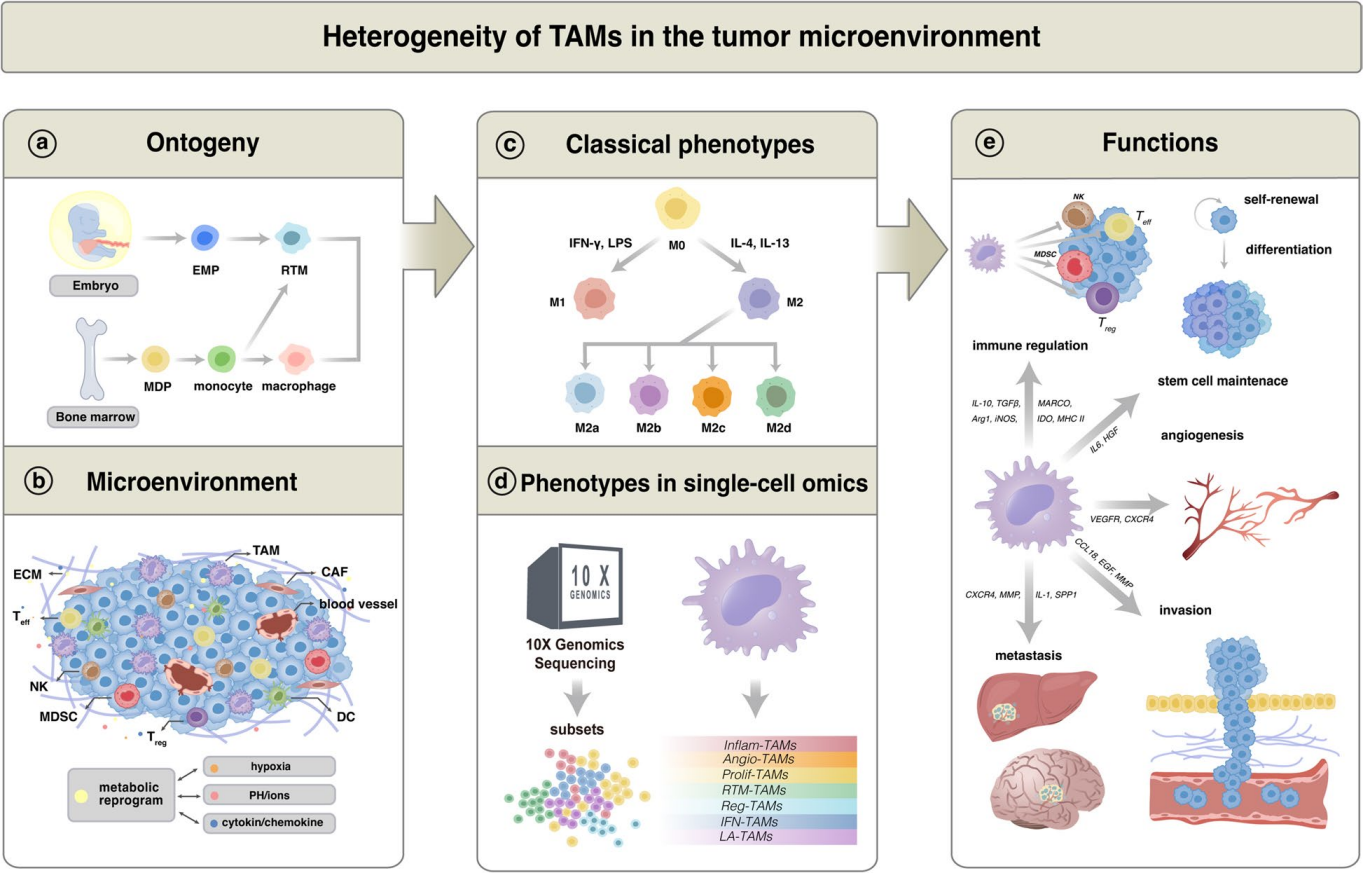
Heterogeneity of TAMs in tumor microenvironment witnessed in the era of single-cell omics. **a**,**b** Single-cell multi-omics underscore TAMs' diverse origins, phenotypes, and functions shaped by the tumor microenvironment, highlighting metabolic reprogramming's influence on TAM heterogeneity. **c**,**d** Beyond the traditional polarization dichotomy, Ruo-Yu Ma et al. classified seven TAM subtypes characterized by specific gene signatures and functions using single-cell omics, named: interferon-stimulated (IFN-TAMs), immune-modulated (Reg-TAMs), inflammatory cytokine-enriched (Inflam-TAMs), lipid-associated (LA-TAMs), pro-angiogenic (Angio-TAMs), RTM-like (RTM-TAMs), and proliferative TAMs (Prolif-TAMs). **e** TAMs' heterogeneity is linked to their varied functions, with emerging research clarifying their roles in tumor progression. *MDP* Myeloid differentiation primary response protein, *RTM* Resident tissue macrophages, *EMP* Erythromyeloid progenitors, *Treg* Regulatory T cells, *MDSC* Myeloid-derived suppressor cells, *Teff* Effector T cells, *NK* Natural killer cells, *ECM* Extracellular matrix, *CAF* Cancer-associated fibroblasts, *HGF* Hepatocyte growth factor, *CCL18* Chemokine (C–C motif) ligand 18, *EGF* Epidermal growth factor, *MMP* Matrix metalloproteinase, *CXCR4* C-X-C chemokine receptor type 4, *SPP1* Secreted phosphoprotein 1, *VEGFR* Vascular endothelial growth factor receptor, *MARCO* Macrophage receptor with collagenous structure, *Arg1* Arginase 1, *iNOS* Inducible nitric oxide synthase, *IDO* Indoleamine 2,3-dioxygenase

The enrichment of TAMs in the microenvironment of tumors is a well-established hallmark, with these cells constituting a significant proportion of the inflammatory cells present in the tumor stroma [13]. A growing number of single-cell sequencing data suggest that TAM exhibits significant variability not only across various cancer patients but also between various malignant lesion locations within the same patient and within a specific tumor lesion [14–16]. Studies in mice and clinical trials indicate that the quantity and characteristics of TAMs in various tumor areas—including areas with a substantial tumor cell density (referred to as tumor "nests"), the perivascular niche, and poorly vascularized, hypoxic/necrotic tumor regions—correlate with survival and free of recurrence among human tumors [14]. This implies that TAMs in various tumor regions have unique origins, sensitivities to local signals, and capacity to either promote or inhibit tumor development and responsiveness to therapy. Through single-cell atlas comparison, Che et al. found higher TAM heterogeneity in the TME of colorectal liver metastases without preoperative chemotherapy. In contrast, this diversity, especially among less mature and less activated TAMs, was reduced in tumors treated with preoperative chemotherapy [16]. Remarkably, the era of single-cell analysis has illuminated the heterogeneity of TAMs, as demonstrated through their diverse origins, phenotypes, and functional roles (Fig. 1).

### The origins of TAMs

With the development of lineage tracing techniques, TAMs of different origins have been verified to exhibit distinct phenotypes and functions [17, 18]. TAMs can originate from both circulating Ly6C^+^CCR2^+^ monocytes and resident tissue macrophages (RTMs) close to the tumor, as has been demonstrated in the context of gliomas and pancreatic malignancies (Fig. 1a) [19, 20]. Significantly, it should be noted that the RTMs that can be co-opted into TAMs also represent a heterogeneous population. Studies over the last 20 years have refuted the common idea that RTMs are continually resupplied by blood monocytes produced from bone marrow (BM) precursors [12]. With Cre-loxP-based inducible fate mapping models, these studies have provided evidence of local macrophage proliferation within tissues, indicating the presence of embryonically derived macrophages capable of self-preservation independent of circulating progenitor cells (Fig. 1a) [21–23]. Notably, both BM-derived and embryonically derived macrophages contribute variably and dynamically to the RTM population across different tissues [12]. The establishment of monocytic and granulocytic tracing models via Ms4a3^TdT^, Ms4a3^Cre^, and Ms4a3^CreERT2^ mice has affirmed that most macrophages in several tissues, including the brain, epidermis, liver, and lungs, are derived from the embryonic hematopoietic system [24]. These macroph ages are seeded during embryonic development and maintain their populations through self-renewal, as evidenced by microglia in the brain, Langerhans cells in the epidermis, and Kupffer cells in the liver [24]. Adult BM-derived monocytes can only develop into RTMs of certain specific tissues, such as macrophages in the intestine and dermis [12, 24]. Moreover, in various states such as infection, injury, and tumors, embryonic-derived RTMs are continually diminished. Circulating monocytes are recruited to these tissues, where they differentiate into monocyte-derived macrophages, fulfilling crucial functions [24]. Therefore, owing to the heterogeneity of their origins, there is some inter-tumor heterogeneity in the extent to which RTMs are involved in the progression of different tumors (Fig. 1a).It was found that the proportion of the two origins varied in different carcinomas, along with dynamic changes in the temporal and spatial distribution. RTMs-derived TAMs accumulate in the vicinity of tumor cells during early tumor formation and promote epithelial-mesenchymal transition and invasiveness of tumor cells, whereas during tumor growth, RTMs redistribute at the periphery of the TME, which becomes dominated by monocyte-derived macrophages in both mouse and human NSCLC [25]. Further, in breast cancer, as the tumor progresses, the number of TAMs produced by RTMs converts gradually decreases, while the number of TAMs generated by BM-derived monocytes increases simultaneously [26]. In this case, decreasing RTMs does not affect tumor growth, while ablating classical monocytes inhibits tumor progression. However, in pancreatic cancer, contrary to breast cancer, RTMs proliferate during tumor progression and acquire a transcriptional profile favorable to pancreatic cancer fibrosis; reduction of BM-derived TAMs does not affect this process but can be reversed by disruption of RTMs [27]. Furthermore, using a spectral tracking approach, it could be determined that this population of RTMs-derived TAM was generated from embryonic macrophages rather than from adult bone marrow monocytes, expanded by in situ proliferation in PDAC, and showed significant pro-fibrotic activity that accelerated the progression of PDAC [28]. The investigation that embryo-derived tissue-resident CD163^+^ Tim4^+^macrophages, rather than bone marrow-derived macrophages, promote metastatic spread of ovarian cancer provides another evidence supporting the idea that different sources of TAMs play distinct roles in tumor progression [29, 30].

To sum up, the aforementioned observations underscore the pivotal role of ontogeny in shaping the diversity of TAMs. Concurrently, the high-dimensional data procured from single-cell omics and the in-vivo lineage tracing models impart a deep understanding of the crucial interplay among the TAMs' origin, phenotype, and function.

### The phenotypes of TAMs

Based on the inflammatory function of TAMs, two activation phenotypes of macrophages have been widely studied: M1 (classically activated) and M2 (alternatively activated) (Fig. 1c) [31]. M1 macrophages, responsive to various stimuli like IFN-γ, LPS, or TNF-α, exhibit high antigen presentation and complement-mediated phagocytosis, contributing to an inflammatory response [31]. Their pro-inflammatory activities help neutralize foreign substances and hinder tumor formation [31]. Alternatively, M2 macrophages are categorized into M2a, M2b, M2c, and M2d based on their activating factor [32]. M2a and M2b primarily perform immunomodulatory roles, supporting Th2-mediated responses, while M2c and M2d function in immune suppression and tissue remodeling [32]. Notably, all M2 macrophages produce IL-10, an immunosuppressive cytokine critical for tumor growth [32]. However, the conventional dichotomy of M1 and M2 may not fully cover the phenotypes of macrophages in vivo. Recent research has revealed that macrophages incrementally adopted attributes of the M2 phenotype, yet without substantial diminution of the M1 signature, which demonstrated the limitations of the traditional classification system [33, 34]. This has prompted a renewed interest in the field and the development of new experimental approaches and nomenclature frameworks that better reflect the heterogeneity of TAMs.

Significantly, recent scRNA-seq data suggest that TAMs often co-express mature M1 and M2 markers and that phenotypically and functionally different TAM subpopulations coexist in TMEs (Fig. 1d; Table 1) [15, 16, 35, 36]. Indeed, single-cell multi-omics approaches, which incorporate transcriptomic, epigenomic, and metabolomic information, have significantly benefited our understanding of the molecular variety of TAMs [15, 16, 35, 37]. Such studies have clustered TAM subgroups according to commonalities, revealing the heterogeneity of TAMs and their distinct subpopulations exhibiting different functions and behaviors. Several large-scale pan-cancer single-cell RNA sequencing studies have identified unique molecules in TAMs, showing a far larger variety of expression-specific molecules than previously considered to be conceivable. Even though different researchers use diverse nomenclatures, there is growing consensus about the kinds of TAMs at the transcriptome level, which is crucial for integrating multiple studies and extending our knowledge of TAMs heterogeneity. A recent review of scRNA-seq cancer studies noted that certain subsets of TAMs are retained in almost all cancer types, and Ruo-Yu Ma et al. classified these TAMs into seven subgroups based on their signature genes, enrichment pathways, and predicted functions, naming them interferon-stimulated (IFN-TAMs), immune-modulated (Reg-TAMs), inflammatory cytokine-enriched (Inflam-TAMs), lipid-associated (LA-TAMs), pro-angiogenic (Angio-TAMs), RTM-like (RTM-TAMs), and proliferative TAMs (Prolif-TAMs) (Fig. 1d; Table 1). Ruo-Yu Ma et al. do point out, however, that this list of TAM subgroups is far from complete or exclusive, and that the transcriptome variety of TAMs in all cancer types may resemble a continuous spectrum rather than separate parts. Because of this dynamic variety of TAM subgroups, it is challenging to generalize using a static signature.

**Table 1:** Classification and characterization of TAM subtypes from M1/M2 to single-cell sequencing insights

*TAM subtypes*		*Signature*	*Cancer types*	*Factors Secreted by TAMs*	*Functions*	*Ref.*
*Traditionally defined populations*	M1	CD80, CD86, CD68, MHC-II, iNOS, TLR-4	LC	IL-12, IL-6, TNF-α	Pro-inflammatory, tumor resistance	[38]
	M2a	CD163, CD206	CC	IL-1R, IGF, TGF-β, CCL17, IL-10	Anti-inflammatory, tissue remodeling	[39, 40]
	M2b	CD86, TNF	LC	IL-10	Immunoregulation, tumor progression	[38, 40]
	M2c	CD163, CD206, TLR-1, CCR2	Melanoma	TGF-β, IL-10	Phagocytosis of apoptotic cells	[32, 40]
	M2d	CD86, iNOS	lymphoma	VEGF, IL-10	Angiogenesis, tumor progression	[32, 40]
*Major populations defined by scRNA-seq insights*	SPP1^+^	SPP1, PMAIP1, INHBA, KLF2/6, NEDD9, G0S2	CRC, OS, GC, OC	CCL2/3/4, CXCL1/2/3/5/8, IL-6	tumor angiogenesis, Recruiting immune cells	[11, 16, 36, 41, 42]
	FOLR2^+^	FLOR2, CD163, CD206, TIM4	HCC, BRCA, LUAD, OC	CCL17/19/22	CD8 + T-cell infiltration, Tregs interaction, immunosuppression	[11, 43–45]
	TIE2^+^	TIE2, VEGFR, CCR2, CXCR4	OC, BRCA	CCL2, VEGF, MMP7	Metastasis, angiogenesis tumor progression	[46–48]
	TREM2^+^	TREM2, ZEB1, FABP5, CD163, CD36, CD63, AOPE, APOC1	CRC, OS, GC, NSCLC, BRCA	CCL18, LPL, MMP7/9/12, SPARC	Lipid metabolism, immunosuppression, matrix remodeling	[37, 41, 42, 49, 50]
	MARCO^+^	MARCO, Arginase, MHC-II, MRC1	CRC, BRCA, GC, HCC	Arginase, IL-10, CCL22	Immunoregulation, tumor progression	[49, 51]
	FCN1^+^	FCN1, FLT1, FN1, CEBPB, CD163, CD52, CXCR4, TIMP1, VCAN	CRC, GBM, BRCA, HCC, OC	CCL2/4/20, IL1B, IL1RN, IL8, MIF, VEGFA	Angiogenesis, tumor progression	[11, 41, 43, 45, 52]
	C1QC^+^	C1QC, C1QB, C1QA, APOE, TREM2, GPNMB, SLCO2B1, APOC1, RNASE1, AXL	BRCA, OS, UCEC, CRC	C1QC, C1QB, C1QA, APOE, APOC1, CCL2/8, CXCL8/10	Phagocytosis, tumor progression	[41, 50, 51, 53]
	ISG15^+^	ISG15, IFITM3, GBP1, IL1RN	CRC, BRCA, GC	CCL2, CXCL10	Pro-inflammatory	[49–51]

TAMs display both M1 and M2 markers, challenging the adequacy of the M1/M2 dichotomy. scRNA-seq uncovers a richer diversity of TAM subtypes with varied phenotypes and functions cohabiting the tumor landscape. *LC* Lung Cancer, *CC* Cervical Cancer, *SPP1* Secreted Phosphoprotein 1, *CRC* Colorectal Cancer, *OS* Osteosarcoma, *GC* Gastric Cancer, *OC* Ovarian Cancer, *FLOR2* Folate Receptor 2, *HCC* Hepatocellular Carcinoma, *BRCA* Breast Cancer, *LUAD* Lung Adenocarcinoma, *TIE2* Tyrosine Kinase with Immunoglobulin-Like and EGF-Like Domains 2, *TREM2* Triggering Receptor Expressed On Myeloid Cells 2, *UCEC* Uterine Corpus Endometrial Carcinoma, *MARCO* Macrophage Receptor With Collagenous Structure, *FCN1* Ficolin 1, *GBM* Glioblastoma Multiforme, *PDAC* Pancreatic Ductal Adenocarcinoma

Thus, in the era of single-cell multi-omics, understanding the phenotypic diversity of TAMs is crucial. This diversity, invariably linked to the array of TAM functions in tumor progression, underscores the complexity of tumor dynamics and challenges the oversimplified dichotomous view of TAM roles.

### The function of TAMs in tumor progression

An elevated presence of TAMs is correlated with an unfavorable prognosis in diverse cancers, this finding is attributable to their tumor-supportive characteristics such as promoting angiogenesis, instigating immunosuppression, and facilitating cancer cell dissemination [1, 37]. TAMs regulate angiogenesis in the tumor microenvironment through the release of pro-angiogenic substances vascular endothelial growth factor (VEGF), basic fibroblast growth factor (bFGF), and chemokines (CXCL8 and CXCL12), and anti-angiogenic substances like TSP-1. Therefore, they can both promote and inhibit angiogenesis, depending on specific circumstances [54]. Moreover, TAMs can promote tumor progression by inhibiting immune responses. They express T-cell immune checkpoint ligands, attract Tregs, and produce immunosuppressive molecules like IDO, TGF, and IL-10, impairing cytotoxic T cells and natural killer cells' functions [55, 56]. By releasing IL-4 and IL-13, they also hinder the proliferation of dendritic cells [57]. Furthermore, TAMs stimulate enzymes, such as matrix metalloproteinases (MMPs), facilitating matrix breakdown, thereby enabling tumor cell motility and invasiveness, and fostering metastasis [58, 59]. They also produce chemokines like CXCL12 and CCL2, attracting tumor cells to distant sites [60].

We now have a much better grasp of the variety of TAM on several levels with the advent of single-cell multi-omics, and more functional investigations are already underway (Fig. 1e) [61]. As an illustration, LYVE1^+^RTMs are linked to the suppression of inflammation and fibrosis in colorectal cancer (CRC) [41], while SPP1^+^TAMs are linked to tumor angiogenesis in CRC [41], and FOLR2^+^TAMs support onco-fetal reprogramming of TME in HCC [43]. Moreover, the roles recruited and resident macrophages play may vary based on the specific malignancy. In a mouse model of pancreatic cancer, local macrophages generated from embryos multiply in situ and display pro-tumorigenic behavior, unlike their bone marrow counterparts, which mostly display an antigen-presenting phenotype and may therefore act as an anti-tumor agent. Meanwhile, subpopulations of differentiated TAMs may be differentially regulated and have differential pro-tumorigenic capacity [12]. For example, our prior studies indicate that in epithelial ovarian cancer, Tie2^+^TAMs recruited to tumor ascites by Angiopoietin-2 (Ang2) enhance endothelial function and tumor angiogenesis via IGF1-induced signaling [46]. Further, the upregulation of Regulated in Development and DNA Damage Responses 1 (REDD1) allows TAMs in hypoxic environments to prevent glycolysis and promote tumor metastasis [9, 62].

However, the relationship between TAMs and cancer prognosis is not always straightforward and can yield inconsistent findings. For instance, high TAM densities have been correlated with favorable prognosis in some cancers like NSCLC, HCC, and ovarian cancer[1]. Indeed, whereas certain TAM subpopulations promote carcinogenesis, angiogenesis, immune escape, and therapeutic resistance that are eventually linked to poor disease outcomes, other TAM populations carry out tumoricidal actions that assist the effectiveness of many anticancer therapies [42, 63]. For instance, Zhou et al. reported a marked infiltration of pro-inflammatory FABP4^+^macrophages in lung metastases of osteosarcoma, with key favorable prognostic implications [42]. These tumoricidal TAMs may aid in identifying and eliminating cancer cells through the production of cytotoxic agents like Nitric Oxide (NO) and ROS [64].

Undoubtedly, the diversity of TAMs is increasingly appreciated in current research. Over the past decade, studies have focused on identifying the molecular processes that underlie the phenotypic and functional variability of TAMs. Investigating the genesis of TAM diversity is crucial for a comprehensive understanding of the complexity of these potent immune cells in cancer, and for the development of precise diagnostic and therapeutic approaches.

## Factors shaping the heterogeneity of TAMs

The heterogeneity of TAMs arises from a combination of factors, including both intrinsic cellular origins and extrinsic environmental cues in the TME, particularly those generated from intercellular communications. The functional heterogeneity of TAMs induced by their origins is progressively coming into focus because of the new paradigm of macrophage ontogeny, which has shown that the TAM pool in tumor tissues is made up of both bone marrow-derived and embryonic macrophages. Data from a series of single-cell sequencing reveals that origin heterogeneity corresponds to its transcriptional profile and phenotype heterogeneity [65]. Bone marrow myeloid progenitor cells produce monocytes that leave the circulation and further differentiate into macrophages in tissues. The differentiation and migration of BM-derived macrophages are regulated by CSF1R and its ligands IL-34 and CSF1 [12, 14, 66]. In addition, the recruitment of BM-derived monocytes and their differentiation into TAMs requires the activation of a series of integrins. Cytokines and chemokines in TME, such as CSF1, GM-CSF, IL-1β, CCL2, and VEGF, can induce conformational changes in α4β1 integrins to promote myelopoiesis, monocyte-to-tumor trafficking, and infiltration by activating the corresponding signaling pathways [14, 67]. In particular, IL-1β, SDF1α, and VEGF in the TME activate G-protein coupled receptors (GPCRs), receptor tyrosine kinases (RTKs), and TLR/IL-1R, thereby activating Ras and its downstream target PI3Kγ [68]. Activation of PI3Kγ amplifies the signaling cascade, inhibits the activation of NF-κB and promotes c/EBPβ-mediated signal transduction, which mediates the production of TAMs [68]. Inhibition of this signaling cascade reduces monocyte recruitment and TAM aggregation in tumors. Embryonic antecedents (yolk sac or fetal liver), also undergo differentiation in tissues and their derivative macrophages become RTMs [69]. RTMs are subjected to soluble factors and additional factors in TME and are domesticated into pro-tumor growth TAMs, which also promote the recruitment of BM-derived macrophages. This indicates that ontogeny significantly influences the diversity of TAMs. As previously elucidated, distinct progenitors contribute to the manifestation of various phenotypes and functions, highlighting the complexity of TAM dynamics.

While the innate origins and plasticity of macrophages do contribute to heterogeneity among TAMs, microenvironmental signals within the TME also shape TAM phenotype, function, and sustainability in a tumor-specific manner. Previous literature often presumed that macrophages from a single tissue make up a homogenous population, while a couple of recent investigations have revealed evidence indicating the presence of sub-tissular niches, by which heterogeneous macrophage populations dwell [70, 71]. The macrophages that reside there are profoundly and mysteriously shaped by the surroundings. Even though the same embryonic-derived liver RTM, both CD206^−^ and CD206^+^subpopulations still exist in Kupffer cells, as demonstrated [71]. This contradiction between CD206^−^ and CD206^+^populations, completely irrespective of the origin of the macrophages, might be defined by the established ecological niche, in accordance with the idea that the ecological niche is a dominating element in the concept of macrophage features [71].

Indeed, aberrant physicochemical features in the TME play an essential role in the heterogeneity of TAMs. For instance, multiple ion channels are expressed abnormally in the TME [72]. Scientists have revealed that high K^+^ions in tumor tissues restrict the plasticity of TAMs, and inhibiting the Kir2.1 potassium channel induces TAM metabolic reprogramming and repolarizes M2-like TAMs to a tumor-killing M1-like state [72]. Furthermore, elements such as chemotherapeutic stress, nutrient availability, oxygen tension, and surrounding pH can reconfigure TAMs, thereby affecting their diverse phenotypes and functions [73].

The acidification of the TME, featuring elevated levels of lactate, arginase, and VEGF, is one of the factors that impact the phenotypic and functional heterogeneity of TAMs [74, 75]. In conditions like murine melanomas, LLC lung adenocarcinoma, and colon carcinomas, the enhanced glycolysis of cancer cells leads to GPCR activation on TAMs. These receptors, which sense TME acidification, contribute to the M2-like polarization of TAMs [75, 76]. Correspondingly, a study elucidated how tumor cells and macrophages interact through the lactate-Gpr132 axis. Upon sensing lactate abundance in the TME, macrophage G-protein coupled receptor 132 (Gpr132) polarizes into M2-like macrophages, a process that promotes metastasis and invasion in breast cancer. The inhibition of Gpr132 has been shown to reduce the metastasis of breast cancer [76]. Although lactate has traditionally been considered as an energy substrate and metabolic waste, its non-metabolic regulatory role in macrophage phenotypes is also significant. Recent investigations have shed light on lactylation, a process involving lactate-derived modifications of histone arginine residues, as an epigenetic alteration that directly initiates chromatin gene transcription [77, 78]. Utilizing bacterially-challenged M1 macrophages as a model system, a study revealed an "intrinsic lactate timer" that activates gene expression to promote homeostasis, particularly during the late phase of M1 macrophage polarization [77]. This discovery of histone lactylation augments our comprehension of lactate's functionality and its contribution to oncological conditions. Subsequent studies have found that metabolic reprogramming and epigenetic regulation of TAMs in response to an acidic tumor microenvironment are closely related [78]. Notably, the lactate-induced polarization of TAMs towards an M2-like phenotype depends on ATP Citrate Lyase (ACLY) activity and mitochondrial pyruvate uptake, potentially regulating M2-like gene transcription, histone acetylation, and immune suppression via lactate-dependent Tricarboxylic Acid (TCA) cycle[78].

In tandem with lactate, the hypoxic TME induces HIF-1α stabilization within TAMs. This hypoxic state significantly influences TAM phenotype and functionality by instigating a metabolic shift from oxidative processes to glycolysis, mediated by HIF-1α stabilization [74, 79]. Notably, oxygen content is closely associated with vascular distribution and blood perfusion. Extracellular metabolite gradients produced by tumor cells significantly influence the functional and phenotypic diversity of TAMs in the TME, guiding their differentiation based on ischemia levels and spatial location[54]. TAMs incorporate hypoxia cues from the tumor environment into the gradual activation of MAPK signaling, generating predictable gene expression patterns. These phenotypic changes have significant functional implications [54]. For instance, ischemic macrophages induce tube-like morphogenesis in adjacent endothelial cells, replenishing blood flow in nutrient-starved, angiogenesis-dependent regions. Similarly, in the autogenous mouse PyMT mammary tumor model, distinct metabolic variations occur between normoxic and hypoxic regions of the vessel, affecting TAMs' phenotype: pro-inflammatory in normoxia and anti-inflammatory in hypoxia [54]. Concordantly, another study investigating the heterogeneity within TAMs has revealed a more compact accumulation of anti-inflammatory macrophages within particularly hypoxic regions within tumors [80]. Moreover, M2-like TAMs were more abundant in the perinecrotic zone of glioblastoma and high-grade glioma (HGG) [81, 82]. TAM in a hypoxic environment favorably regulate HIF-1α expression and boosts IL-8 production, which in turn stimulates PD-L1 expression, encouraging the growth and spread of esophageal cancer cells [83].

Throughout different stages of tumor progression, the phenotypic and functional heterogeneity of TAMs can be influenced by varying cytokines within the microenvironment, manifesting as a co-evolutionary interaction between TAMs and tumor cells [84]. Pro-inflammatory cytokines generated by tumor cells help recruit and polarize M1-like TAMs early in the carcinogenesis process, eliciting a variety of anticancer effects [85]. At this stage, M1-like TAMs may specifically produce cytotoxic substances such as NO and ROS to destroy tumor cells, engage in phagocytosis, and release pro-inflammatory cytokines, thereby further stimulating anti-tumor immunity [85]. Nevertheless, when the tumor grows, the tumor cells repolarize to M2-type TAMs by a variety of pathways, including the production of cytokines including CSF-1, IL-4, and IL-10, lactate secretion, nutrient shortage, and hypoxia [86]. In turn, M2-like TAMs facilitate tumor angiogenesis by secreting growth factors such as VEGF and TGF-β; immunosuppressive factors like IL10; factors that activate the self-renewal and proliferation of tumor stem cells, such as IL-6 and STAT3; and proteases that alter the extracellular microenvironment, like matrix metallopeptidases, to encourage tumor progression [86].

It is established that TAMs are highly sensitive to their environmental niche. They continually take cues from their immediate surroundings, alter their identities, and adjust their functions as a result. Thus, it is challenging to simplify since there are many interconnected levels involved in the process of comprehending TAM's variability and intricacy. Among the plethora of microenvironmental stimuli, specific metabolic alterations inherent to the TME and the subsequent metabolic reprogramming of TAMs are crucial precipitators of their functional and phenotypic heterogeneity. Additionally, the metabolic regulation of TAMs exhibits extensive interplay with various other elements within the microenvironment. This complex interaction, sculpted by the TME, will be comprehensively examined and elucidated in the following discourse.

## Metabolic reprogramming of TAM in the tumor microenvironment: The driver of heterogeneity and functional plasticity

It has been abundantly clear in recent years how many facets of cellular function are supported by metabolism and how metabolic reprogramming may influence cell differentiation and destiny [87]. Similar to the “Warburg effect” observed in tumor cells, the phenomenon of metabolic switching in immune cells such as macrophages after activation, to meet increased energy demands and biosynthesis, is referred to as “immunometabolism”, implying that these metabolic adaptations directly affect immune cell function by regulating transcriptional and post-transcriptional events, beyond simply providing energy to support immune activity in specific circumstances [88, 89].

Multiple investigations have scrutinized the intracellular signaling reactions, unique pathways in specific cell types, potential activities, and functions of significant metabolic pathways at the transcriptome level of single cells. Notably, different metabolic processes are present in TAM clusters, as shown by pathway analysis of the scRNA-seq data. Meanwhile, accumulating research is revealing a strong correlation between the phenotypic characteristics and the metabolism of TAMs [49]. These studies have revealed that metabolism can alter the phenotypic and functional alterations of TAMs in response to changes in the tumor microenvironment.

### Glucose metabolism

**Fig. 2:**
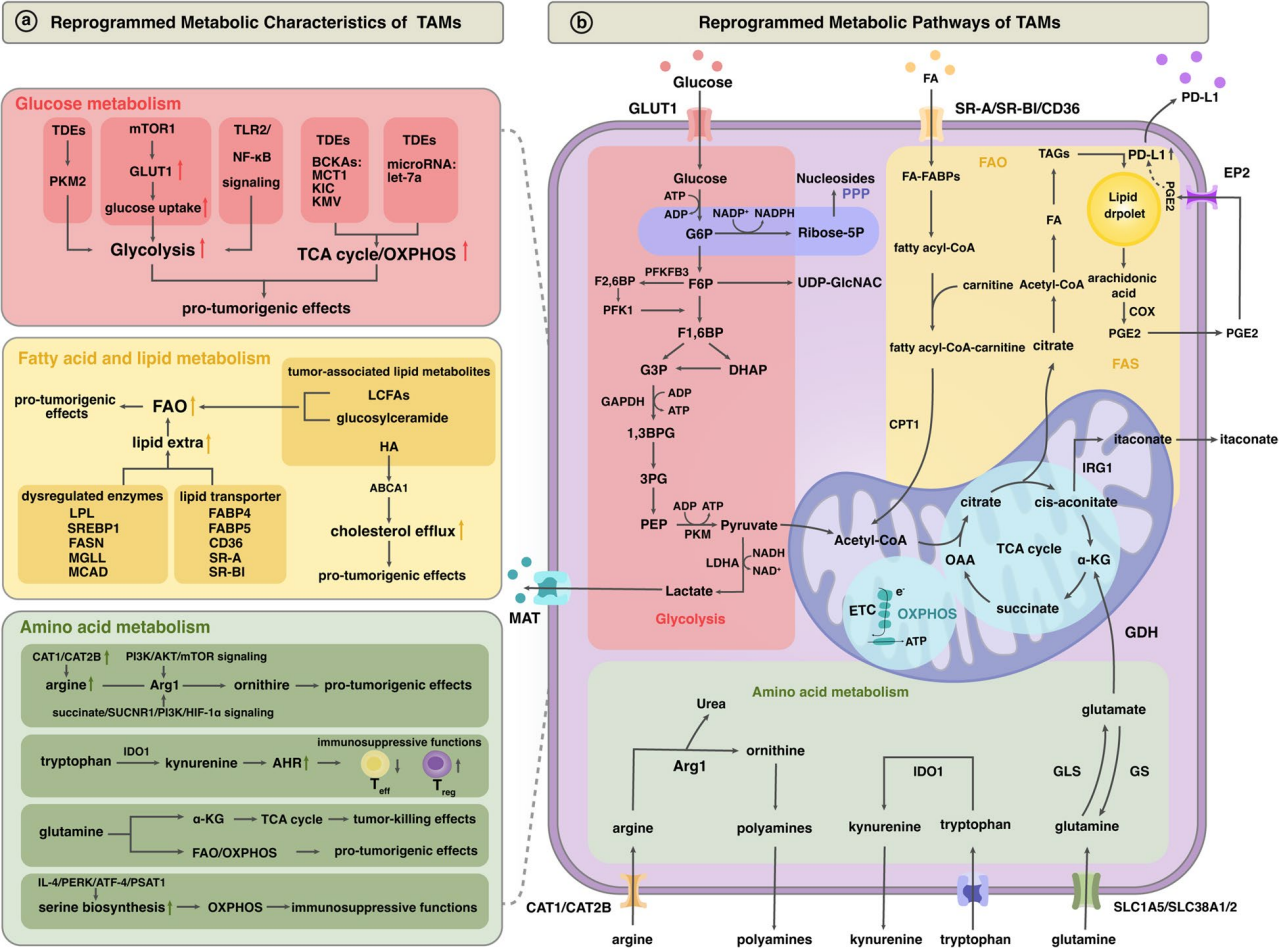
Metabolic reprogramming of TAM in the tumor microenvironment. **a** TAMs exhibit distinct metabolic characteristics compared to BMDMs, with increased glucose and fatty acid metabolism, supporting their immunosuppressive and tumorigenic activities. They show enhanced glycolysis, TCA cycle, and OXPHOS, along with FAO and cholesterol efflux. Further, as they respond to microenvironmental cues, the unique metabolic pathways in TAMs, particularly those involving specific amino acids, underpin their functional heterogeneity. **b** The interconnected metabolic pathways of TAMs, driven by tumor environment cues, contribute to their functional and phenotypic diversity. Key components include GLUT1, LDHA, and enzymes and transporters involved in lipid and amino acid metabolism. *BCKAs* Branched-chain keto acids, *MCT1* Monocarboxylate transporter 1, *KIC* Ketoisocaproic acid, *KMV* Ketomethylvaleric acid, *PKM2* Pyruvate kinase M2, *GLUT1* Glucose transporter 1, *LCFAs* Long-chain fatty acids, *SR-BI* Scavenger receptor class B type 1, *MCAD* Medium-chain acyl-CoA dehydrogenase, *MGLL* Monoglyceride lipase, *FASN* Fatty acid synthase, *SREBP1* Sterol regulatory element-binding protein 1, *FABP* Fatty acid-binding protein, *LPL* Lipoprotein lipase, *ABCA1* ATP-binding cassette transporter A1, *HA* Hyaluronic acid, *AHR* Aryl hydrocarbon receptor, *PERK* Protein kinase R, *ATF-4* Activating transcription factor 4, *PSAT1* Phosphoserine aminotransferase 1, *G6P* Glucose-6-phosphate, *DHAP* Dihydroxyacetone phosphate, *PFK1* Phosphofructokinase-1, *F2,6BP* Fructose 2,6-bisphosphate, *PFKFB3* 6-Phosphofructo-2-kinase/fructose-2,6-biphosphatase 3, *UDP-GlcNAc* Uridine diphosphate N-acetylglucosamine, *F6P* Fructose 6-phosphate, *F1,6BP* Fructose 1,6-bisphosphate, *G3P* Glyceraldehyde 3-phosphate, *1,3BPG* 1,3-Bisphosphoglyceric acid, *3PG* 3-Phosphoglyceric acid, *PEP* Phosphoenolpyruvate, *GAPDH* Glyceraldehyde 3-phosphate dehydrogenase, *PKM* Pyruvate kinase M, *LDHA* Lactate dehydrogenase A, *TAGs* Triacylglycerols, *FA* Fatty acid, *Acetyl-CoA* Acetyl coenzyme A, *CPT1* Carnitine Palmitoyltransferase1, *FA-FABPs* Fatty acid-Fatty acid-binding proteins, *CAT2B* Cationic amino acid transporter 2B, *GLS* Glutaminase, *GS* Glutamine synthetase, *GDH* Glutamate dehydrogenase, *SLC1A5* Solute carrier family 1 member 5, *SLC38A1/2* Solute carrier family 38 members 1 and 2, *OAAs* Oxaloacetates

In the traditional sense, macrophages respond to microenvironmental cues, like inflammation and injury, with classically M1 and M2 types displaying metabolic heterogeneity [90]. M1 macrophages utilize glycolysis, exhibiting TCA cycle interruptions that enhance HIF1α stabilization and glycolysis [91]. Conversely, M2 macrophages rely on a complete TCA cycle and oxidative phosphorylation (OXPHOS)[90]. However, under the remodeling of the tumor microenvironment, the glycometabolism of TAMs exhibits increasingly complex and diverse characteristics (Fig. 2).

#### Glycolysis

As a metabolic pathway for the conversion of glucose to pyruvate, glycolysis is essential for the immunological activity of macrophages, despite its low energy yield. During phagocytosis or inflammation, macrophages display increased glycolysis, which correlates with M1-like polarization, characterized by increased IFN-γ and 6-phosphofructo-2-kinase/fructose-2,6-biphosphatase 3 (PFKFB3)[92]. Inhibiting PFKFB3 curbs IFN-γ-induced glycolysis, suppressing M1 polarization [92]. The conversion of glucose to pyruvate via GLUT-1 and then to acetyl coactivators via the TCA cycle favor the transcription of pro-inflammatory genes and the release of inflammatory mediators, hence the typically high rate of glycolysis in M1 macrophages [93]. The regulation of glycolysis in M1 macrophages relies on several critical signaling pathways and transcription factors, including the inflammatory TLR/NF-κB pathway, PI3K-Akt pathway and so on [94, 95]. Classically, M2 macrophages prefer OXPHOS and enhanced fatty acid metabolism for tissue remodeling, wound healing and anti-inflammation, with lower glycolytic activity compared to M1 [93].

Incorporating comprehensive multi-omics assessments at the individual cellular and subcellular scales disclosed that TAMs show substantial diversity in their glucose metabolic processes, as well as in their phenotype and function across diverse tumor contexts [96]. In certain tumor circumstances, TAMs undergo a metabolic shift towards oxidative metabolism and reduced glucose intake. This leads to hyperactivation of endothelial cells, promoting neoangiogenesis and metastasis in the tumor microenvironment due to increased glucose availability [74]. Consequently, decreased glycolytic activity in TAMs promotes tumor progression through both nutritional and immunological mechanisms. In most cases, however, the metabolic adaptation to hypoxic TME enables TAMs to engage in glycolysis, providing them with the required energy and biosynthetic resources to support their pro-tumoral functions. Indeed, the metabolic reliance on glycolysis in anti-inflammatory TAMs stands in stark contrast to cultured non-tumor related bone marrow-derived macrophages (BMDMs), underscoring the multifaceted intricacy of macrophage polarization in the in vivo setting [97]. Studies have surprisingly revealed that TAMs display the most substantial glucose uptake, followed by T cells and tumor cells, all of which heavily rely on the functional activity of mTORC1 [98, 99]. The TME is distinguished by robust nutrient competition, attributed to the exclusive expression of the M2 isoform of pyruvate kinase (PKM2), in tumor cells [100, 101]. Our previous study has revealed that upregulation of PKM2 expression in ovarian cancer cells can enhance cellular glycolysis and chemoresistance by promoting the expression of c-Myc [102]. Notably, Brioschi et al. demonstrated that exosomes carrying PKM2 from HCC enhance glycolysis in TAMs, increasing acetyl-CoA production [22]. Consequently, histone acetylation and STAT3 phosphorylation are triggered in the nucleus, upregulating transcription factors linked to M2-like phenotype differentiation [22]. Moreover, the secretion of cytokines/chemokines by TAMs, via the CCL1-CCR8 axis, strengthens the PKM2-ARRDC1 interaction in HCC. This reinforces PKM2 production in HCC cells, establishing a feedback loop that promotes tumor initiation [22]. Consequently, in the tumor microenvironment, glycolysis uniquely becomes essential for M2-like polarization of TAMs, unlike non-tumor BMDMs. Inhibition of glycolysis with 2-deoxy-D-glucose (2-DG) inhibited the polarization of M2-like TAMs via an AMPK-HIF-1α-dependent pathway [103]. Meanwhile, tumor acidosis, a result of the high metabolic activity and poor perfusion within tumors, stimulates the glycolytic pathway in TAMs while concurrently suppressing oxidative metabolism [104]. Despite exhibiting elevated glycolytic activity, this metabolic shift promotes the pro-tumoral M2-like phenotype in TAMs and contributes to immune evasion and tumor progression [104]. These investigations suggest that tumor development might be accelerated by TAM glycolysis, even amidst heightened competition for local glucose availability (Fig. 2).

Specifically, TAM metabolism undergoes a unique alteration of glycolysis, driven in part by the action of nitric oxide synthase 2 (NOS2), which produces NO, aiding in the activation of TAMs and promoting M2-like polarization [93]. Meanwhile, tumor-derived exosomes (TDEs) play a pivotal role in reorienting TAMs towards an immunosuppressive, glycolytic-dominant metabolic phenotype, a process orchestrated through the TLR2 and NF-κB pathway. This pathway not only facilitates increased glucose uptake but also stimulates further NOS2 production [105]. Notably, a positive feedback loop is established between elevated glycolysis and TAM's immunosuppressive effects. Findings have indicated that glycolysis' intermediate metabolites, such as lactate, trigger M2-like polarization and anti-inflammatory effects by activating G protein-coupled receptor 81 (GPR81), also known as Hydroxycarboxylic acid receptor 1 (HCA1) [106]. This is accompanied by augmented lactate production, which bolsters PD-L1 expression and reinforces the immunosuppressive qualities of CD206^+^ PD-L1^+^TAMs [105]. In a compelling twist, recent research has unveiled that IL-4, commonly associated with anti-inflammatory M2 polarization, can provoke pro-inflammatory M2 macrophages [107]. This is achieved by amplifying glycolytic metabolism through the Wdr5/H3K4me3 axis, thereby resulting in a more pronounced pro-inflammatory phenotype in trained macrophages via elevated glycolytic metabolism and HIF-1α stabilization [107]. Nevertheless, it remains to be seen whether this also plays a role in the metabolic regulation of TAM by the TME.

#### TCA cycle and OXPHOS

Several cellular constituents within the TME engage in extensive metabolic interactions with TAMs (Fig. 2). Notably, the crosstalk in glycometabolism between TAMs and tumor cells reprograms TAMs through the TCA cycle and OXPHOS, contributing to the immunosuppressive microenvironment. Tumor cells can reprogram the TAM metabolism by secreting metabolites to reshape the microenvironment, or by directly regulating the TAM metabolizing enzymes. Recent research has revealed that specific branched-chain ketoacids (BCKAs) released by distinct human and mouse cancer cells can significantly impact macrophage metabolism and phenotype in a monocarboxylate transporter protein 1 (MCT1)-dependent manner [108, 109]. Mechanistically, α-ketoisocaproic acid (KIC) and α-keto-β-methylpentanoic acid (KMV) promote macrophage polarization towards a pro-tumorigenic state by facilitating TCA cycle intermediates and polyamine metabolism. Conversely, α-ketoisovaleric acid (KIV) exhibits a pro-inflammatory effect on macrophages [108]. In a murine tumor model, using fluorescent B16ZsGreen cells, researchers noted metabolic and phenotypic changes in non-resident macrophages during early metastasis due to the uptake of tumor-derived microparticles [110]. These particles swiftly activated mTORC1 in macrophages, increased mitochondrial mass, and prompted metabolic programs enhancing OXPHOS, paralleled by an upregulation of VCAM1, CD38, and CD63, aiding anti-metastatic function in pre-metastatic lungs [110]. Intriguingly, this anti-metastatic phenotype appears to diminish over time, likely due to prolonged exposure to growing metastatic lesions and the emerging immunosuppressive tumor microenvironment [110]. However, the microparticles' composition remains uncharacterized, warranting future research to explore if they contain damaged mitochondria influencing macrophage metabolism and the impact of macrophage reprogramming during metastasis. At the same time, another study showed that the presence of microRNA let-7a within TDEs hampers mTOR signaling and glycolysis in TAMs, consequently promoting enhanced OXPHOS and the upregulation of M2-like markers, namely CD206 and arginase-1 (Arg1) [111].

Besides, itaconic acid (ITA) exemplifies the immune metabolic reprogramming of macrophages in the TME (Fig. 2). In response to stimuli such as LPS, TLR, and IFN I/II cytokines, immune response gene 1 protein (IRG1) expression is upregulated in macrophages, which encodes aconitate decarboxylase (ACOD1) that catalyzes the production of ITAs and redirects the use of cis-aconitate from the TCA cycle towards itaconic acid production [112]. A study has shown that itaconate was one of the most strongly elevated metabolites in the peritoneal RTMs in peritoneal tumors, including B16 melanoma or ID8 ovarian carcinoma, and itaconate exhibited a pro-tumor effect, as proven by the dramatically decreased peritoneal tumors after Irg1 knockdown [113]. This process includes increasing OXPHOS with itaconate, which in turn boosts ROS generation and accelerates tumor development [113]. In line with this, recent research has shown that tumor cells induce Irg1 expression in macrophages by activating the NF-κB pathway, and the resulting ITAs inhibit the expression of inflammatory genes and the infiltration of CD8^+^T cells into tumor sites [114]. Further, oncogenes such as MYC and KRAS may promote the infiltration of BMDMs and contribute to their differentiation into PD-L1^+^ VEGF^+^ CD206^+^TAMs, as shown in mouse models of lung cancer [115]. In accordance with this, a study integrating transcriptomic and metabolomic analyses revealed that pancreatic cancer cells with *Kras*mutations secrete GM-CSF, resulting in metabolic dysregulation and polarization of TAMs [116]. GM-CSF activates the PI3K/AKT pathway in TAMs, upregulating ACLY activity and promoting citrate breakdown [116]. Additionally, GM-CSF upregulates enzymes involved in the TCA cycle, including Arg1, leading to elevated levels of immunosuppressive metabolic byproducts such as ornithine [116]. Moreover, a recent study has identified a crucial function of the TCA enzyme, fumarate hydratase (FH), in macrophages. Stimulation with LPS or inhibition of FH causes a reconfiguration of the TCA cycle within macrophages. This change elevates fumarate levels, which in turn inhibits mitochondrial respiration and generates a potent inflammatory response. Intriguingly, inhibition of FH can augment the secretion of interferon-β through mechanisms involving mitochondrial RNA (mtRNA) release and activation of RNA sensors such as Toll-like receptor 7 (TLR7), retinoic acid-inducible gene (RIG)-I, and melanoma differentiation-associated protein (MDA)5 [117]. These observations underscore the protective role of FH in maintaining appropriate macrophage cytokine and interferon responses. This role may be particularly relevant to the pathogenesis and treatment of diseases associated with reduced FH levels, such as Systemic Lupus Erythematosus (SLE) and FH-deficient renal carcinoma [118]. Nonetheless, further research is required to decode the specific mechanisms through which FH regulation in TAMs within the pan-cancer microenvironment influences their inflammatory effects.

Indeed, the investigation of single-cell multi-omics has unveiled the curtain on the metabolic reprogramming of TAMs within the TME. Researchers have identified population heterogeneity in the TME-driven metabolic reprogramming of TAMs. At the single-cell transcriptomic level, several studies have now identified TAM subpopulations undergoing metabolic reprogramming associated with glycolysis. For instance, different TAM subsets show certain metabolic characteristics in human NSCLC and the 3LL-R Lewis lung cancer mouse model [119]. Specifically, TAMs with high MHCII expression exhibit a hampered TCA cycle, whereas those with low MHCII levels demonstrate increased oxidative and glycolytic metabolism [119]. Additionally, these MHCII-low TAMs boost L-arginine metabolism, predominantly utilize lactate as a carbon source, and improve their ability to inhibit T cells [119]. Further, the single-cell atlas of human glioblastoma has shown that the spatial activation of macrophages within the TME is associated with the ontogeny and metabolic variations of TAMs [120]. Remarkably, the microenvironment acts differently on the metabolic reprogramming of the two groups of cells, blood-derived TAMs did not generally exhibit the metabolic phenotype of tissue-resident microglia; instead, this population of TAMs raised the M2-like markers of immunosuppressive cytokines and oxidative metabolism [120]. Interestingly, in the ovarian cancer model, Tim4^+^ TAMs display greater levels of OXPHOS and respond to mitosis to reduce oxidative stress as compared to Tim4^−^TAMs [30]. Additionally, Tim4^+^ TAMs produce more Arg1, which decreases arginine levels and inhibits mTORC1 even more. Due to variations in their metabolism, only Tim4^+^ TAMs, not Tim4^−^TAMs, can promote the peritoneal metastasis of ovarian cancer [30]. Also, TME is altered during immune checkpoint therapy (ICT), which affects TAM activation and differentiation through metabolic reprogramming. In the later stages of ICT, CD206^+^TAMs show increased expression of genes involved in OXPHOS, a change that corresponds to the regulation of specific markers that characterize macrophages (such as CD206) [121]. However, there are still large gaps in the mechanisms by which the regulation of glycolysis by TME in specific subgroups leads to functional and phenotypic heterogeneity, and further research is needed.

### Fatty acid and lipid metabolism

Generally, macrophage lipolysis is linked to immunosuppression, while lipid synthesis is connected to inflammation, as demonstrated by studies [92]. Within the TME, upregulated fatty acid oxidation (FAO) can promote mitochondrial OXPHOS and ROS generation. This, in turn, may phosphorylate JAK1, activate STAT6, initiate downstream gene transcription, and promote M2-like polarization of TAMs, ultimately facilitating tumor invasion [122]. Macrophages internalize various lipids via endocytosis or receptors like CD36, SR-A, and SR-BI, leading to subsequent metabolic pathways influenced by environmental stimuli (Fig. 2b) [123]. TAMs have been shown to utilize distinct exogenous fatty acids from the TME, influencing their pro-tumoral functions. Meanwhile, some TAMs accumulate intracellular lipids, supporting their metabolic functions and the modulation of the immune response (Fig. 2a) [124].

The TME triggers a remodeling of lipid metabolism in TAMs through the dysregulation of several lipid-metabolizing enzymes (Fig. 2a). These encompass Lipoprotein Lipase (LPL), Sterol Regulatory Element-Binding Protein 1 (SREBP1), and Fatty Acid Synthase (FASN), showcasing a comprehensive modification in metabolic activities. Monoacylglycerol lipase (MGLL) deficiency, in particular, induces lipid accumulation in TAMs, enhances CB2/TLR4-dependent macrophage activation, and subsequently hinders the function of CD8^+^T cells associated with tumors and impedes the development of various cancers [124]. Additionally, the fatty acid-binding protein (FABP) family is renowned for its role in intracellular fatty acid binding and transport. Differential expression of FABPs was observed in TAMs across various stages of breast cancer [125]. TAMs in advanced breast cancers exhibited preferential FABP4 expression, promoting tumor growth via IL6/STAT3 signaling [125]. In contrast, TAMs infiltrating early-stage cancers more commonly expressed FABP5, associated with lipid droplet production and release of immunostimulatory cytokines like IFN I [125]. However, further investigations are warranted to elucidate the microenvironmental mechanisms regulating these differential FABP expressions. Medium-chain acyl-CoA dehydrogenase (MCAD), an enzyme involved in fatty acid β-oxidation, exhibits significant dysregulation in TAMs within the TME. Notably, compelling findings indicate that in certain in vitro and in vivo contexts, MCAD inhibition occurs due to caspase1-dependent cleavage of the peroxisome proliferator-activated receptor γ (PPARγ) [126]. Caspase1, usually activated by immunostimulatory stimuli, forges a connection between these stimuli, its activation, and the metabolic implications tied to MCAD inhibition [127]. This illuminates potential circuitry wherein immunostimulation within the TME could trigger compensatory immunosuppression through metabolic modifications of TAMs.

Moreover, tumor-associated lipid metabolites affect the composition of the TME and the function of intra-tumoral macrophages (Fig. 2a). It was found that certain types of Long-Chain Fatty Acids (LCFAs) released from tumor cells in the metastatic microenvironment were transported by extracellular vesicles, shaped a lipid-rich TME, and functionally reprogrammed macrophages by upregulating CD36, which then upregulated FAO activity in macrophages and promoted their polarization from M1-like to M2-like phenotype [128]. In this situation, TAMs accelerate tumor growth by activating the PPARδ to release the immunosuppressive cytokine IL-10 [129]. In contrast, blocking CD36 in metastasis-associated macrophages with inhibitors restored immunity of CD8^+^T cells and improved liver metastasis in preclinical mouse models [128]. Moreover, the heightened expression of lipid metabolism genes within the TME, such as TIAM2 in Lung Adenocarcinoma (LUAD), is linked to the polarization of M2-like TAMs, signifying a pivotal role in the immunosuppressive environment [130]. Concurrently, another study suggests that the absence of Receptor-Interacting Protein Kinase 3 (RIPK3) incites both the tumor invasion and M2-like polarization of TAMs, achieved through the alteration of fatty acid metabolism within the TME. Importantly, this study demonstrates that hindering FAO effectively reverses the immunosuppressive properties of TAMs and curbs the progression of Hepatocellular Carcinoma (HCC) [131]. Further, the study indicates that glucosylceramide, originating from tumor cells, instigates an unorthodox endoplasmic reticulum (ER) stress reaction within TAMs [132]. This leads to a modification in the lipid structure and saturation on the ER membrane. Such changes stimulate IRE1-mediated spliced XBP1 generation and STAT3 activation, collectively amplifying the protumorigenic characteristics and the expression of immunosuppressive genes [132]. Hyaluronic acid (HA), secreted by ovarian cancer cells, facilitates cholesterol efflux in TAMs through binding to CD44 [133]. This leads to diminished intracellular cholesterol levels in TAMs, subsequently activating IL-4R/STAT6/PI3K signaling [133, 134]. Mechanically, the PI3K/Akt pathway is involved in upregulating ABCA1, an ATP-binding cassette transporter that facilitates cholesterol efflux. Additionally, cholesterol efflux diminishes lipid rafts within TAMs, impairing IFNγR signaling and suppressing the expression of pro-inflammatory genes, including IL-12 and iNOS [133]. Notably, targeting cholesterol efflux genes (ABCA1 and ABCG1) or inhibiting IL-4R/STAT6/PI3K has been shown to significantly impede ovarian cancer progression in preclinical models [133]. PGE2 is a dual-effect inflammatory factor. Notably, due to enhanced arachidonic acid metabolism in certain TAM subsets and the activation of apoptosis processes in tumor cells responding to therapy, PGE2 is generated inside the tumor microenvironment [73]. In addition to PKM2 and HIF1α, a signal transduction cascade launched by PGE2 controls the transcriptional level of PD-L1 expression in TAMs [135, 136]. Moreover, PGE2 exerts pro-tumorigenic effects by stimulating the migration of macrophages to the TME and polarization to an M2-like profile in certain contexts, along with boosting cancer cell proliferation [137].

Besides, in combination with single-cell sequencing and other techniques, several studies have identified specific groups of lipid metabolic reprogramming in TME, and the metabolic profile of these groups has been correlated with immunosuppressive and tumor-promoting functions. Recent research identified two subpopulations of lipid-associated macrophages (LAM1 and LAM2) in human breast cancer by combining CITE-seq with 10X Visium ST and scRNA-seq [37]. These subpopulations exhibited high expression of the fatty acid metabolism genes FABP5 (LAM1) and APOE (LAM2), respectively, and both were present in invasivecancer areas, whereas LAM2 was also present in areas with high stromal, adipocyte, lymphocyte and high PD1/PD-L1 staining [37], suggesting that these highly lipid metabolically active TAMs are associated with their potent immunosuppressive function. Intriguingly, single-cell sequencing of a mouse model of lung metastasis from breast cancer also revealed a subpopulation of TAMs with a profile consistent with lipid-associated macrophages, marked by genes such as Lgals3 and Trem2 [138]. These TAMs not only exhibited significantly reduced phagocytic ability but also showed enrichment of genes related to lipid metabolism, extracellular matrix remodeling, and immune suppression pathways [138]. Furthermore, single-cell analyses of early-stage smoking-associated Non-Small Cell Lung Cancer (NSCLC) patients uncovered two distinct immunosuppressive TAM subsets within the NSCLC TME, each demonstrating unique functional metabolic profiles [50]. Specifically, CCL18^+^macrophages, characterized by high levels of fatty acid oxidative phosphorylation metabolism, exert immunosuppressive effects by stifling the production of inflammatory factors [50]. In contrast, SPP1^+^macrophages predominantly utilize glycolysis, a process that propels tumor metastasis by stimulating angiogenesis and matrix remodeling [50]. However, the criteria for selecting these unique subsets, the prevalence of subsets characterized by aberrant lipid metabolism, and the complex mechanisms by which the TME governs lipid metabolic reprogramming within these subsets are still not fully understood. Consequently, these aspects necessitate a deeper and more comprehensive exploration.

### Amino acid metabolism

In the context of amino acid metabolism, the TME plays a crucial role in shaping TAM functionality. Broadly, the diverse metabolic pathways of specific amino acids exert significant influence on the immune activity of macrophages. For instance, the metabolic processing of arginine in macrophages significantly dictates their tumor-promoting/anti-inflammatory or tumor-suppressing/pro-inflammatory phenotypes (Fig. 2) [91, 139]. As evidence suggests, Arg1 facilitates the conversion of arginine into ornithine and urea, substances known to promote tumor growth, while concurrently curtailing the synthesis of tumoricidal NO [140]. Indeed, the immunosuppressive phenotype observed in TAMs of both mice and humans is molded by the arginase pathway, which generates ornithine and polyamines, along with inhibitory cytokines like IL-10 [141, 142]. The activation of the PI3K/Akt/mTOR signaling pathway in the tumor microenvironment upregulates Arg1 expression in TAMs [143]. Notably, Arg1 expression in TAMs and tumors can be inhibited with PI3Kγ inhibitors, and PI3Kγ deletion promotes the development of NOS, which may accelerate the creation of tumor-killing NO from L-arginine [141, 144]. As a result, blocking PI3Kγ is a viable tactic for controlling metabolic alterations that support immune activation and cancer prevention. Remarkably, TAMs demonstrate enhanced expression of cationic amino acid transporters 1 and 2B (CAT-1 and CAT-2B) in comparison to myeloid cells unrelated to tumors, thereby facilitating augmented arginine uptake and consequent depletion of arginine within the TME [140, 145]. Meanwhile, the findings suggest that upon immune attack, tumor cells metabolize arginine to generate asymmetric dimethylarginine (ADMA), which hinders NO synthesis by inhibiting NOS enzyme activity. This manipulation of macrophage metabolism and polarization promotes tumor tolerance [146]. Furthermore, employing scRNA-seq analysis in conjunction with experimental validation, researchers have demonstrated that a population exhibiting high expression of macrophage scavenger receptor 1 (MSR1) manifests M2-like polarization characteristics through the reprogramming of arginine and proline metabolism. This activation induces the AMPK/mTOR pathway, promoting the progression of gastric cancer [147]. However, whether the reprogramming of arginine metabolism is a characteristic of a specific subset or subsets of TAMs, as well as the mechanisms by which the microenvironment regulates arginine metabolism in these populations, remain to be further investigated.

In addition to arginine metabolism, the TME also modifies tryptophan metabolism in TAMs, contributing to their immunosuppressive phenotype (Fig. 2). The enzyme IDO catalyzes the initial step of the kynurenine pathway, converting tryptophan to kynurenine, reducing tryptophan levels, accumulating the immune-suppressing amino acid kynurenine, and encouraging the establishment of Treg cells [148, 149]. Interestingly, tumor overexpression of IDO1 is associated with the amplified assembly of TAMs characterized by high expression of CD206, secretion of elevated levels of TGFβ, and a decrease in pro-inflammatory macrophages expressing NOS2, CD86, and IL-12 within the TME [148]. This manipulation of TAMs appears dependent on the induction of arylhydrocarbon receptor (AHR) expression through IDO induction within tumor cells [148, 150]. Glioblastoma induces the upregulation of AHR expression in TAMs through the secretion of kynurenine [150]. This, in turn, promotes the expression of CCR2, facilitating TAM recruitment in response to CCL2 signaling [150]. Additionally, AHR activation drives the expression of KLF4 and suppresses NF-κB activation in TAMs [150]. Simultaneously, selective AHR blockade can slow down the progression of tumors with high expression of IDO and TDO by reshaping the TAMs. Furthermore, when used in combination with PD-1 blockade, its therapeutic efficacy is enhanced [148]. The AHR signaling cascade also stimulates the transcription of cytochrome p450 enzymes, including CYP1A1, which play significant roles in the metabolism of polyunsaturated fatty acids [151]. Nevertheless, further extensive research is required to elucidate the distinct functions of these enzymes in the metabolic activities of TAMs at the cellular level.

It is well known that glutamine addiction occurs in tumor cells, while glutamine deprivation in the TME also affects the metabolism and behavior of TAMs (Fig. 2). In clear cell renal cell carcinoma, glutamine consumption by tumor cells results in local glutamine deprivation in the extracellular space, which activates HIF-1α to trigger TAM secretion of immunosuppressive IL-23, promoting Treg proliferation and the expression of IL-10 and TGF-β [152]. Macrophages exhibit varied functionality and phenotype due to differential metabolic pathways of glutamine. Glutamine degradation in the TCA cycle stimulates inflammatory responses, resulting in a tumor-killing phenotype. Conversely, anti-inflammatory and pro-tumor macrophages rely on glutamine metabolism to promote FAO and OXPHOS. In cultured BMDMs, glutamine drives the polarization towards an M2-like phenotype and enhances the transcription of key M2-associated genes such as Arg1 and those involved in UDP-GlcNAc synthesis. This process occurs through epigenetic modifications, including the demethylation of Jmjd3 [153]. Corroborating these observations, gene eradication of glutamine synthetase in TAMs results in the emergence of more M1-related attributes, such as amplified expression of MHCII and a decreased incidence of CD206^+^TAMs [154].

Moreover, tumor-derived succinate triggers the reprogramming of peritoneal macrophages and enhances their migration via the succinate receptor 1 (SUCNR1)/PI3K/HIF-1α signaling pathway, ultimately increasing Arg1 expression and promoting arginine metabolism [155]. The activation of SUCNR-1 also leads to upregulation of VEGF expression through the activation of ERK1/2 and STAT3, inducing tumor angiogenesis [156]. Furthermore, chronic inflammatory signals in the tumor microenvironment, such as IL-4, can enhance the immunosuppressive functions of TAMs by regulating the metabolism of certain amino acids [157, 158]. For instance, studies have found that the PERK-PSAT1-serine pathway is crucial for promoting M2-like polarization and immune-suppressive function of TAMs in the TME [157, 158]. Mechanistically, IL-4 activates the PERK signaling pathway, which subsequently upregulates PSAT1 via ATF-4. This leads to increased serine biosynthesis, enhancing mitochondrial function and α-ketoglutarate production in TAMs. Inhibition of PERK inhibits immunosuppression and enhances the efficacy of immune checkpoint inhibitors in melanoma [157, 158]. Additionally, as an important source of one-carbon units, amino acid metabolism engages in significant crosstalk with purine metabolism. TAMs with highly active amino acid metabolism can thus provide substrates for purine metabolism. Data from scRNA-seq of mouse glioma Act-MG suggest that microglia-derived TAM gene expression is enriched in “cytoplasmic translation”, while monocyte-derived TAM is enriched in “purine monophosphate metabolism” [65]. Meanwhile, one study verified that this metabolic patterns of TAMs correspond with their functional features, and that enhanced purine metabolism is typical of TAMs with pro-tumor and terminal differentiation phenotypes. They also revealed that purine metabolic characteristics are linked with patient prognosis and responsiveness to ICB [159]. However, whether this terminal subgroup of TAMs characterized by purine metabolism has commonalities, what crosstalk exists with other metabolic pathways such as amino acid metabolism, and the specific mechanisms by which TME shapes them remain to be investigated.

Undoubtedly, single-cell level analyses provide further clues as to the specific microenvironmental factors that shape the metabolism, phenotype, and function of TAMs. For example, single-cell transcriptome analysis of TAMs from mice with hepatic metastatic tumors indicates that hepatic macrophages can be divided into five metabolic clusters characterized by lipid metabolism, purine metabolism, amino acid metabolism, OXPHOS, and glycolysis [159]. Importantly, the different metabolic clusters differ in their antigen-presenting, phagocytic, angiogenic, and immunosuppressive activities[159]. In particular, TAMs with glycolysis have the highest phagocytic capacity, TAMs with purine metabolism have the lowest antigen-presenting capacity, and TAMs with tumor-inducing metabolic features, including purine metabolism and amino acid metabolism, express high levels of pro-angiogenic genes [159]. Additionally, researchers identified a cluster of similar TAMs in PDAC and ovarian cancer, both of which highly express hypoxia-related metabolic genes and are associated with pro-tumor angiogenesis [160, 161]. These observations propose that TAMs in similar metabolic states often express analogous functional gene patterns, irrespective of their ontogenic development, indicating that regulatory factors within the microenvironment play a crucial role in the metabolic reprogramming and phenotypic-functional heterogeneity of TAMs. In harmony with prior discussions, an array of single-cell sequencing data reveals the integration of multiple metabolic modes within TAMs. TAMs exhibiting different metabolic modes possess distinct phenotypes and functions, thus the classification of TAMs based on metabolic modes may be more precise, ultimately benefiting targeted and precision therapies.

## Targeting heterogeneous TAM metabolism: therapeutic potential in cancer immunotherapy

In the past decade, remarkable strides have been made in investigating anti-cancer immunotherapies that target TAMs [162]. Currently, five primary categories of macrophage-directed interventions have emerged: blocking monocyte recruitment; eliminating immunosuppressive macrophages; loading chimeric antigen receptor (CAR) macrophages (CAR-Mac); inducing macrophage reprogramming; and tapping the anti-tumor function of TAMs [33, 66, 163]. The strategy of blocking monocyte recruitment aims to deter the migration of monocytes to tumor sites, thereby forestalling their transformation into TAMs [164]. The use of tools such as TREM2 inhibitors to eliminate immunosuppressive macrophages selectively seeks to restore regular immune responses and stifle tumor growth [165]. The technique of loading CAR-Mac involves the use of genetically modified CAR-Mac to specifically target tumor cells, thereby augmenting the power and precision of anti-tumor immune responses [166]. The tactic of inducing macrophage reprogramming, such as with CLEVER-1 inhibitors, recalibrates TAMs from a pro-tumor to an anti-tumor phenotype [162, 167]. Lastly, the approach of tapping the anti-tumor function of TAMs, exemplified by SIRP1α–CD47 inhibitors, leverages the innate anti-tumor functionalities of certain TAM subsets to impede or halt tumor growth [168].

Significantly, the heterogeneity of TAMs, as unveiled by high-dimensional data, presents both hurdles and opportunities for TAM targeting. While therapeutic objectives can be more effectively achieved by directing efforts toward specific phenotypic and functional subgroups, the inherent complexity and heterogeneity pose challenges to their identification and subsequent targeting. Recent years have seen substantial advancements in elimination strategies, attributed largely to a more profound understanding of TAM heterogeneity. This includes tactics such as obstructing the CCL2-CCR2 axis to deter monocyte recruitment from the bone marrow to inflamed areas; impeding the CSF1-CSF1R axis to induce TAM apoptosis; or thwarting the CXCL12-CXCR4 and ANG2-TIE2 axes to remove distinct TIE2^+^macrophages vital for tumor angiogenesis [162, 169]. Concurrently, it is worth noting that several TAM reprogramming methodologies are presently under clinical investigation. For instance, creatine functions by suppressing the release of immunosuppressive cytokines like IL-10 and TGF-α and escalating the production of pro-inflammatory cytokines like IL-12 and TNF-β. Similarly, PI3Kγ inhibitors operate by encouraging NF-κB activation while inhibiting C/EBPβ activation in TAMs [141, 170]. Intriguingly, one study investigates these two distinct strategies (removing and reprogramming) to modulate TAMs in an autochthonous PDAC mouse model and shows that depletion of pro-tumorigenic TAMs with anti-CSF1R interferes with the antitumor activity of infused T cells [171]. In contrast, TAM reprogramming with agonistic anti-CD40 monoclonal antibodies increases the intratumoral accumulation and longevity of TCR-engineered T cells and promotes tumor cell apoptosis. These observations underscore the importance of considering the differential impacts of TAM modulation on engineered T cells with previously acquired effector activity [171]. Therefore, this study provides significant insights into the intricate regulation of TAMs in cancer immunotherapy. It highlights the potential relationship between TAM functional heterogeneity and TAM regulation complexity. Anti-CSF1R therapy hinders new TAM accumulation, including potential anti-tumor TAM subgroups, while anti-CD40 therapy alters existing TAMs, encompassing functionally distinct TAM subgroups as well [172]. Indeed, manipulating different TAM groups may yield varying efficacy, thereby potentially explaining the differential effectiveness of pan-targeted TAMs.

**Fig. 3:**
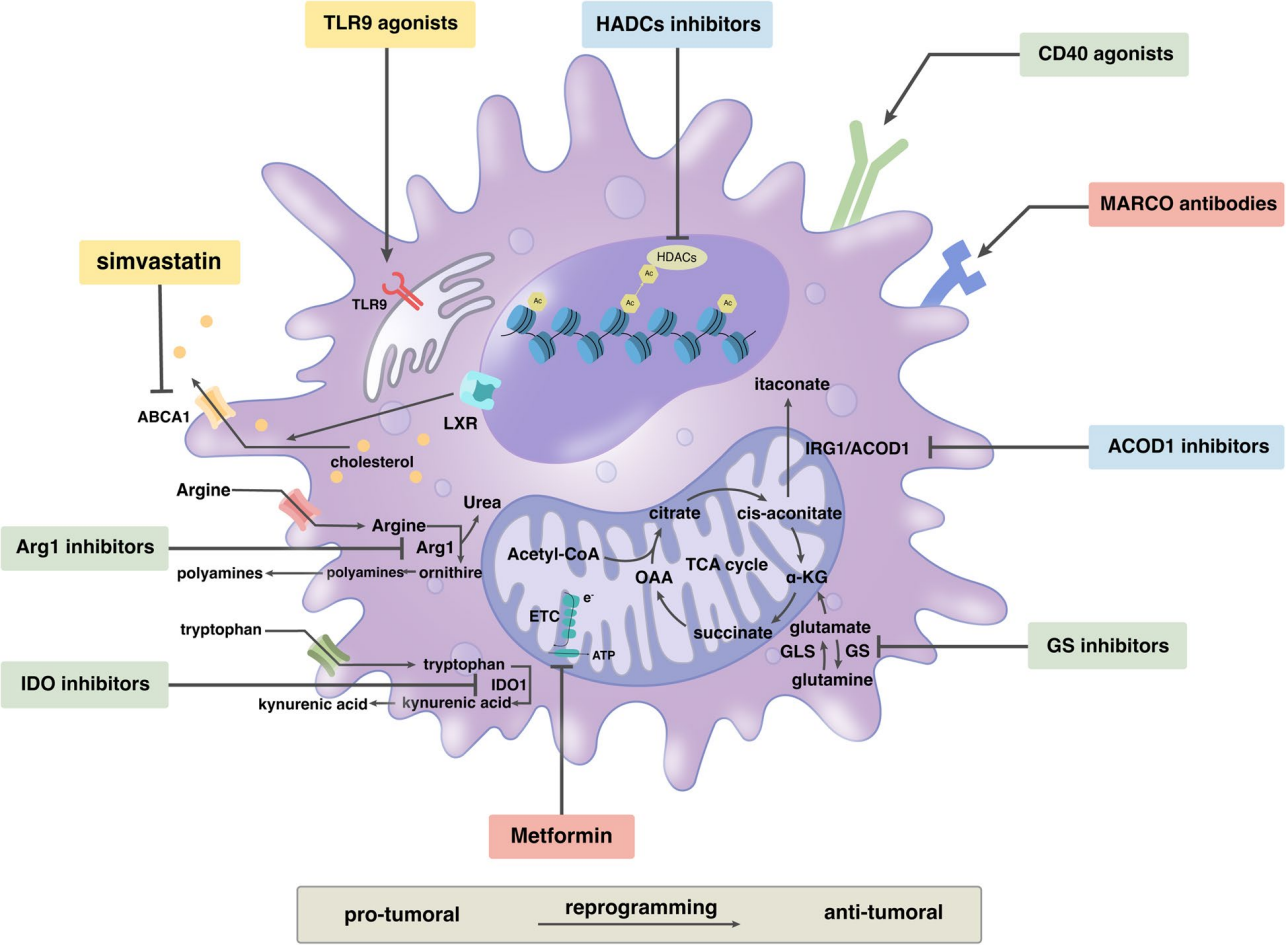
Macrophage metabolic targets and associated Agents. Targeting the metabolic reprogramming of TAMs has the potential to reshape their phenotype and function, thereby offering a promising therapeutic strategy for macrophage repolarization in cancer. The fundamental approach in treating tumors via TAM metabolic reprogramming involves stimulating TAMs towards an M1-like pro-inflammatory phenotype. This strategy includes regulating intracellular ATP levels and redox state, which in turn impacts their phagocytic and digestive abilities and the generation of various signaling molecules, while simultaneously inhibiting M2-like anti-inflammatory polarization. Despite the growing interest in targeting TAM metabolism, our current understanding of TAM metabolic reprogramming is still incomplete, and there remains a dearth of specific targeted therapeutics. Future investigations should focus on exploring the metabolic mechanisms of reprogramming within specific subgroups, in the context of single-cell multi-omics studies, to identify unique therapeutic targets. ETC Electron Transport Chain, *IDO* Indoleamine 2,3-dioxygenase, *GS* Glutamine Synthetase, *LXR* Liver X receptor, *ABCA1* ATP-binding cassette transporter A1, *IRG1* Immune Responsive Gene 1, *ACOD1* Aconitate Decarboxylase 1, *TLR9* Toll-Like Receptor 9, *Arg1* Arginase 1, *HADCs* Histone Deacetylases

Consequently, in the development of TAM-centric strategies, it is paramount to target specific functional TAM subsets, considering the profound impact of their functional heterogeneity on the tumor immune microenvironment. For example, the strategy of targeting CX3CR1^+^TAMs may hold significant potential in impeding metastasis in tumors with an abundance of this subset [173]. This approach will facilitate the creation of targeted therapies by pinpointing which distinct TAM populations are associated with aggressive or resistant malignancies. As previously mentioned, the TME exerts extensive metabolic reprogramming on TAMs. Since this reprogramming is inherent to TAM function and phenotype, it presents a compelling therapeutic tactic for the repolarization of macrophages in cancer. The cornerstone of tumor treatment through metabolic reprogramming of TAMs is to stimulate a transformation of TAMs towards an M1-like pro-inflammatory phenotype. This involves regulating their intracellular ATP levels and redox status, impacting their phagocytic and digestive capabilities, as well as the production of other signaling molecules, while simultaneously suppressing M2-like anti-inflammatory polarization (Table 2; Fig. 3). In subsequent sections, we will delve into several contemporary therapeutic strategies that target TAM metabolism for TAM reprogramming.

**Table 2:** Macrophage metabolic targets and associated therapeutics in preclinical and clinical studies

*Target*	*Drugs*	*Tumor type*	*TAM marker*	*Metabolism*	*Mechanism*	*Type of research/outcome*	*Ref.*
ETC	metformin	the lungs of 4T1 mammary carcinoma mice model	CD11c, CD163	glycolysis, OXPHOS	lower intracellular ATP levels, inhibit the activity of mitochondrial respiratory chain chain, activate the AMPK pathway, promote glucose uptake and glycolysis	preclinical/positive	[174]
ETC	metformin	human esophageal cancer	CD11c, CD163	glycolysis, OXPHOS	lower intracellular ATP levels, inhibit the activity of mitochondrial respiratory chain chain, activate the AMPK pathway, promote glucose uptake and glycolysis	clinical trial phase II	[175]
*MARCO*	MARCO inhibitors, ED31	4T1 mammary carcinoma, MC38 colon cancer carcinoma, the B16 melanoma model NCSLC lung carcinoma model	MARCO, CD68, MHC-II, TIE2	glycolysis	enhance glycolysis	preclinical/positive	[176, 177]
*LXR/ABCA1*	the combination of simvastatin and paclitaxel	A549T lung carcinoma xenograft model	CD206, Arg1, TGF-β	cholesterol metabolism	affect LXR/ABCA1 regulation through cholesterol-related pathways	preclinical/positive	[178]
*TLRs*	TLR 9 agonist, CpG oligodeoxynucleotides	PDAC mice model	CSF1R, Arg1, CD206, MHC-II	lipid metabolism	promote FAO, divert intermediates of the TCA cycle for de novo lipid synthesis	preclinical/positive	[179]
*GS*	GS inhibitor, MSO	LLC lung cancer model	CD80, MHC-II, CD206, CD163	glycolysis, glutamine metabolism	decrease glutamine, increase succinate levels, improve glucose flow via glycolysis	preclinical/positive	[154]
*IDO1*	IDO1 inhibitor, NLG919	B16F10 melanoma model	CD206, CD163	tryptophan metabolism	reverse the M2-like phenotype, the specific mechanisms have not been elucidated	preclinical/positive	[56]
*IDO1*	IDO1 inhibitor, epacadostat	human melanoma	CD206, CD163	tryptophan metabolism	reverse the M2-like phenotype, the specific mechanisms have not been elucidated	clinical trial phase III (NCT02752074)/negtive	[180]
*Arg1*	Arg1 inhibitor, CB-1158	4T1 mammary carcinoma, LLC lung cancer, B16 melanoma, CT26 colorectal carcinoma model	CD68, CD80; CD206, Arg1	argine metabolism	rise in CD80^+^ TAMs and decrease CD206^+^TAMs, the specific mechanisms have not been elucidated	preclinical/positive	[181]
*CD40*	agonistic anti-CD40 monoclonal antibodies, FGK45	YUMM1.7 melanoma model	CD40	FAO, glutamine metabolism	trigger FAO and glutamine metabolism, promote ATP citrate lyase-dependent epigenetic reprogramming of anti-tumorigenic phenotypes in TAMs, glutamine usage reinforces FAO-induced anti-tumorigenic activation	preclinical/positive	[182]

ETC Electron Transport Chain, *AMPK* AMP-activated Protein Kinase, *MARCO* Macrophage Receptor with Collagenous Structure, *LXR* Liver X Receptor, *ABCA1* ATP Binding Cassette Subfamily A Member 1, *TLRs* Toll-Like Receptors, *PDAC* Pancreatic Ductal Adenocarcinoma, *FAO* Fatty Acid Oxidation, *TCA* Tricarboxylic Acid, *GS* Glutamine Synthetase, *MSO* Methionine Sulfoximine, *LLC* Lewis Lung Carcinoma, *IDO1* Indoleamine 2,3-Dioxygenase 1, *Arg1* Arginase 1, *CD40* Cluster of Differentiation 40, *OXPHOS* Oxidative Phosphorylation, *CSF1R* Colony Stimulating Factor 1 Receptor, *NCSLC* Non-Small Cell Lung Cancer, *TIE2* Angiopoietin-1 receptor, *NLG919* Specific inhibitor for IDO1, *epacadostat* Specific inhibitor for IDO1, *CB-1158* Specific inhibitor for Arg1, *FGK45* Specific monoclonal antibody for CD40

### Targeting the metabolic process of TAMs

#### Glucose metabolism

Due to the prominent role of lactate in the TAM immune-suppressive phenotype, targeting glycolysis appears to be a promising strategy for reprogramming TAMs. Most studies that aim to restore macrophage polarization by targeting glycolysis rely on glycolysis inhibitors, such as 2-deoxyglucose (2-DG); however, these inhibitors are far from specific [103]. Since the same glycolytic pathway is also essential for macrophages to combat tumor cell activity, glucose supply is required for ROS generation and phagocytosis [74]. In this targeted strategy, metformin, an anti-diabetic drug, has emerged as a promising candidate (Table 2; Fig. 3). It lowers intracellular ATP levels by inhibiting the activity of mitochondrial respiratory chain complex I, activates the AMPK pathway, and promotes glucose uptake and glycolysis, thereby enhancing pro-inflammatory gene expression within TAMs [183]. In preclinical models, metformin has been shown to promote M1-like differentiation and enhance phagocytic activity by activating the AMPK pathway and inhibiting the STAT3 pathway, thereby reducing the density of immune-suppressive TAMs and reshaping the TME [174]. Relevant clinical trials recruiting patients with different types of tumors are also underway [175]. Additionally, several studies in hepatocellular carcinoma, liver metastases of CRC and NSCLC have detected a similar cluster of TAMs evolving from RTMs with high expression of scavenger receptor MARCO [15, 16, 184], and anti-MARCO treatment was shown to synergize with anti-CTLA4 checkpoint inhibition in the B16 model and MC38 colon cancer mouse model [185]. Aside from inhibiting angiogenesis, studies on the mechanism of action reveal that MARCO inhibitors may cause TAM metabolic reprogramming, including enhanced glycolysis, which transforms TAM into a pro-inflammatory state and activates natural killer cells to mediate tumor death (Table 2; Fig. 3) [176, 177]. Additionally, it is intriguing to note that Mehta et al. discovered that PARP inhibitors can alter the phenotype and function of TAMs through metabolic reprogramming in the BRCA-deficient TNBC mouse model [186]. Utilizing high-dimensional single-cell multi-omics analysis, they found that these inhibitors intensify macrophages' pro- and anti-tumoral properties via alterations in glucose and lipid metabolism, steered by the SREBF1 pathway [186]. Remarkably, integrating PARP inhibitors with CSF1R-blocking antibodies significantly enhanced anti-tumor immunity, thereby prolonging survival in these mice [186]. These observations intriguingly suggest that PARP inhibitors partake in the metabolic regulation of the tumor immune microenvironment. As delineated earlier, microenvironmental cues downregulate FH in macrophages, altering the TCA cycle and reducing mitochondrial respiration. This leads to the release of mtRNA and activation of RNA sensors like RIG-I and MDA5, enhancing interferon-β secretion and inciting a strong inflammatory response in macrophages [117]. The RLR family members, RIG-I and MDA5, functioning as intracellular pattern recognition receptors, are gaining prominence as potential therapeutic targets in cancer due to their capacity to engage with mislocalized mtRNA and other innate RNAs, thus triggering potent immune responses [187]. Emerging research has highlighted the role of the mTOR-LTR-RIG-I axis in various cancers in driving cellular immune responses and facilitating the infiltration of dendritic cells and macrophages [188]. Notably, the overexpression of RIG-I in peritoneal macrophages has been demonstrated to facilitate the transition from M2 to M1 macrophage polarization, thereby impeding tumor advancement [189]. Therefore, the FH-RLR signaling pathway in TAMs emerges as a promising avenue for cancer immunotherapy, albeit necessitating further investigative endeavors to fully elucidate its therapeutic potential.

#### Fatty acid metabolism

TAMs exhibit impaired lipid handling, which is directly associated with the induction of immunosuppressive signaling pathways mediated by the transcription factor LXR, which functions as an oxysterol receptor. Some researchers have found that the combination of simvastatin and paclitaxel can affect LXR/ABCA1 regulation through cholesterol-related pathways, promoting the transition of TAMs from an M2-like to an M1-like phenotype and inhibiting EMT (Table 2; Fig. 3) [178]. However, there are no specific targeted agents for TAMs' LXR and further research is needed. As previously stated, PGE2 plays a key role in the tumor avoidance mechanisms of TAMs related to lipid metabolism [136]. In mouse models, inhibiting prostaglandin G/H synthase 2 (COX2) or using PGE2 receptor EP1/EP2 antagonists can redirect anti-tumor effectors and improve the effectiveness of ICB [190]. Additionally, targeting TAMs in combination with conventional therapy has shown improved therapeutic efficacy. TAM-derived cholesterol has been shown to contribute to therapeutic resistance in prostate cancer [191]. Therefore, researchers have combined TAM-targeted therapy with androgen deprivation therapy (ADT) to limit cholesterol bioavailability and intratumoral androgen production, offering a potential therapeutic approach for patients with castration-resistant prostate cancer (CRPC) [191]. Moreover, recent research has constructed a reduction-responsive RNAi nanoplatform that reprograms tumor lipid metabolism by inhibiting the production of free fatty acids (FFAs) and repolarizes TAMs to a pro-inflammatory phenotype, leading to the secretion of tumor-killing cytokines such as TNF-α and IL-12, which achieves combined anti-cancer effects in both xenografts and in situ pancreatic adenocarcinoma (PAC) tumor models [192]. Intriguingly, researchers have discovered that CpG oligodeoxynucleotides, a TLR 9 agonist, can reprogram macrophage lipid metabolism and activate their antitumor activity (Table 2; Fig. 3) [179]. Mechanistically, CpG can promote intracellular FAO in macrophages and divert intermediates of the TCA cycle for de novo lipid synthesis by activating enzyme carnitine palmitoyltransferase 1A (CPT1A) and citrate lyase. The de novo synthesized lipids, including cholesterol and other lipids, can increase membrane fluidity and enhance the phagocytosis of tumor cells via lipid-mediated engulfment. Therefore, this integrated lipid metabolic pathway can enhance macrophage phagocytosis of CD47^+^tumor cells, providing a potential target for targeted TAM metabolic therapy [179]. As previously highlighted, the scavenger receptor CD36 sees notable upregulation in metastasis-associated macrophages. This receptor enables the endocytosis and metabolism of long-chain fatty acids that originate from tumor cells, thereby strengthening the pro-tumorigenic phenotype of macrophages [128]. Despite the current dearth of studies specifically targeting CD36, these findings underscore its potential as a promising therapeutic target for intervention strategies.

#### Amino acid metabolism

The therapies targeting the amino acid metabolic pathways of TAMs have shown promising results. The dependence of M2-like TAMs on glutamine presents the possibility of targeting glutamine metabolism as a tumor-reprogramming strategy for TAM metabolism. Inhibiting glutamine synthetase (GS) in TAMs can decrease glutamine and increase succinate levels, leading to improved glucose flow via glycolysis and promoting an M1-like phenotype (Table 2; Fig. 3). This shows enhanced T cell recruitment and reduced proangiogenic and pro-metastatic activities of M2-like macrophages in cancer [154, 193]. Recently, the scientific community has vigorously pursued the development of IDO1 inhibitors. Significant progress has yielded small molecule inhibitors, notably epacadostat, indoximod, and BMS-986205, which are presently under clinical trial examination [194–196]. The amplified IDO1 expression in TAMs depletes tryptophan, hampering T cell functionality and fostering immunosuppressive Tregs generation. However, research targeting this pathway has yielded conflicting results (Table 2; Fig. 3) [149]. In preclinical studies, the IDO1 inhibitor NLG919 in combination with Paclitaxel significantly reversed the M2-like TAMs phenotype and the immunosuppressive tumor microenvironment [56]. While in a clinical trial phase III, the combination of an IDO inhibitor epacadostat and pembrolizumab was tested for metastatic melanoma, but the outcome was negative [180]. It is possible that compensatory expression of comparable enzymes, such as tryptophan 2,3-dioxygenase (TDO) and IDO2, may have contributed to this result [149]. Meantime, animal tumors treated with gemcitabine and CB-1158, a potent and orally bioavailable small-molecule inhibitor of Arg1, showed a significant rise in pro-inflammatory (M1-like type, CD80^+^) macrophages and a substantial decline in immune-suppressive (M2-like type, CD206^+^) macrophages (Table 2; Fig. 3) [181]. In line with this, as noted earlier, obstructing Arg1 expression in TAMs via PI3Kγ inhibitors enhances NOS expression, thereby quickening the generation of tumoricidal NO from L-arginine [141, 144]. Consequently, employing PI3Kγ targeting to reconfigure arginine metabolism in TAMs emerges as a promising strategy for tumor treatment. In addition, previous studies have demonstrated that triggering the CD40L-NF-κB pathway in TAMs leads to an increase in the production of pro-inflammatory cytokines and an upregulation of co-stimulatory molecules on the TAM surface [197]. Intriguingly, recent study demonstrated that agonistic anti-CD40 monoclonal antibodies could exert immunotherapeutic effects by coordinating glutamine metabolism and FAO through CD40 signaling, orchestrating epigenetic reprogramming and promoting antitumor polarization in TAMs (Table 2; Fig. 3) [182]. Mechanistically, glutamine metabolism produces lactate and maintains FAO by regulating the NAD^+^/NADH ratio, in contrast to prior observations that glutamine metabolism and FAO promote non-tumor-associated M2 polarization, but instead induce M1-like polarization and antitumor activation of macrophages [182]. Also, the ACLY enzyme, previously associated with epigenetic reprogramming during macrophage polarization, is essential for CD40-mediated M1-like activation [182]. Thus, utilizing agonistic CD40 monoclonal antibodies to activate TAMs and alter the immune response within the TME holds significant promise as a therapeutic approach for cancer patients.

### Targeting metabolites of TAMs

Macrophage metabolic plasticity, controlled by an intricate interplay of metabolic byproducts, transcription factors, and epigenetic modifications, can be strategically harnessed. One promising approach involves epigenetic regulation through the inhibition of Class IIa histone deacetylases (HDACs), which can enhance the expression of MHC Class II and co-stimulatory molecules on TAMs, thereby improving their antigen-presenting capabilities [198, 199]. This intersection of epigenetic regulation and TAM metabolic reprogramming, especially via HDAC inhibition, opens novel avenues for anti-cancer exploration and intervention (Fig. 3). TMP195, a selective HDAC inhibitor, has been reported to reprogram TAMs into cells able to sustain a robust CD8 T cell-mediated anti-tumoral immune response, in an autochthonous mouse model of breast cancer and colorectal cancer [199, 200]. Low-dose HDAC inhibitors, trichostatin-A (TSA) and CG-745, modify the tumor immune microenvironment by reducing TAM suppressive activity and MDSC recruitment, thereby enhancing immunotherapy effectiveness [201, 202]. However, HDAC inhibition also escalates PD-L1 expression, counteracting benefits. Thus, combining low-dose TSA with anti-PD-L1 treatment, which led to tumor reduction and extended survival in mice, suggests a potential strategy to augment anti-tumor macrophage efficacy [201, 202]. Tucidinostat, a selective HDAC inhibitor targeting HDAC1, HDAC2, HDAC3, and HDAC10, is approved for treating advanced, hormone receptor-positive, HER2-negative breast cancer post-endocrine therapy [203]. However, its potential as the first HDAC inhibitor for solid tumor treatment is tempered by its high-grade toxicities, necessitating appropriate dosage determination for effective tumor microenvironment modification [204]. These findings provide a strong rationale for better clinical outcomes in solid tumor patients, but further research is needed before HDAC inhibitors can be considered truly effective in clinical practice.

Besides, IRG1-mediated production of itaconic acid in TAMs can contribute to the immunosuppressive function of TAMs by inhibiting the activation of T cells and NK cells. The growth of various types of tumors is inhibited and the effectiveness of anti-PD-(L)1 immunotherapy is improved in mice with Irg1 deletion [114]. These findings suggest that ACOD1 is a potential target for immune-oncology drugs, and that IRG1-deficient macrophages represent a promising cell therapy strategy for cancer treatment, even in pancreatic tumors that are resistant to T cell-based immunotherapy (Fig. 3). Further, it is interesting to note that MAO-A inhibitors, medications that have been successfully used to treat several kinds of neurological disorders by inhibiting MAO-A's activity in the brain and preventing the breakdown of monoamine neurotransmitters, can also inhibit MAO-A activity in TAMs of human breast cancers and melanomas. This lowers ROS levels and sensitizes the JAK-Stat6 pathway, triggering TAMs to repolarize towards an immunostimulatory phenotype [205]. Consequently, although still in their infancy, these strategies have been demonstrated in animal tumor models to encourage the repolarization of TAMs and have therapeutic anti-cancer effects. More research could be carried out on these targets in the future.

Correspondingly, given the critical impact of metabolic reprogramming on TAM function and phenotype, understanding the metabolic heterogeneity of TAMs from a single-cell multi-omics perspective is of great significance for precise targeting of TAM metabolism [73]. Primarily, the TME is composed of malignant cells and non-malignant components interacting and forming the global metabolic state. Non-targeted drugs affecting shared metabolic pathways (glycolysis, FAO) impact all cell components, making overall effects unpredictable due to potential reductions or counteractions. Further, as previously demonstrated, there is significant metabolic variation not only across distinct TAM subpopulations (e.g., of different origins), but also between subpopulations with similar characteristics (e.g., of the same origin). This might be one factor in the poor effectiveness of existing TAM-targeted treatments [206]. At the same time, the classification of TAM subgroups is still ambiguous. A clearer classification of TAM subpopulations, based on metabolic heterogeneity as a characteristic feature, may be more promising for TAM-targeted therapies. In summary, understanding the heterogeneity of TAMs from a metabolic perspective is of great significance for targeting macrophages and improving anti-tumor efficacy. Yet, a significant research gap persists in targeting TAM metabolism. Future studies should explore TAM metabolic reprogramming and heterogeneity to improve therapeutic efficacy through novel target identification.

## Conclusion and perspective

TAMs are a type of innate immune cell that is frequently observed in high numbers across various cancer types. Their presence is now widely acknowledged as a critical factor in the tumor-associated immune response. TAMs play a crucial role in the response, as their phenotype and function are diverse and can either support or inhibit tumor growth, thereby impacting the efficacy of different treatment modalities. Moreover, since the tumor microenvironment can significantly alter TAMs' phenotype through metabolic reprogramming, it is essential to investigate the evolution of their repertoire and function in response to specific microenvironmental stimuli. Therefore, comprehending the complexity of TAMs is crucial, and it can aid in developing therapies that target specific cellular states or their associated functions.

Studies over the years have shown that there are limitations in classifying TAMs into M1 and M2 types and that heterogeneous TAMs elucidated in the single-cell omics era still lack valid classification criteria. Significantly, the ultimate goal of studying TAM heterogeneity is to determine their function and connect genotype and metabolic phenotype to anticipate patient- and disease-specific outcomes, enabling physicians and scientists to better target specific TAMs for precision therapeutic purposes. The above, along with a series of studies on TAM metabolic reprogramming, indicates that TAMs in the same metabolic state tend to express similar functional gene patterns, regardless of their ontogeny. Genomic and proteomic analyses have suggested that a specific TAM with a certain profile may undergo a particular functional change from an upstream predictive perspective, while metabolomics suggests that a change has occurred from a downstream perspective. Therefore, in the future, characterizing the functional features of TAMs from a metabolic perspective may provide a more accurate basis for their heterogeneity classification. Several studies have already identified specific TAM subgroups based on metabolic heterogeneity, such as Lipid-associated TAMs expressing lipid/fatty acid metabolism-associated genes such as TREM2, FABP5, and APOE in human and murine breast cancer. We speculate that this approach may provide novel insights into understanding TAM heterogeneity and targeting TAM metabolism for therapeutic purposes, and further research is required to characterize the distinct metabolic features of specific subpopulations within the tumor microenvironment and to elucidate their relationships with function and phenotype.

Various experimental methods, including flow cytometry, CyTOF mass spectrometry, NMR, and MS-based metabolomics, contribute to immunometabolism study [207–210]. However, these, especially when used individually, partially characterize cellular metabolic states due to metabolite loss, damage, and limited in vivo analysis [207, 208]. Single-cell RNA sequencing is a cost-effective and comprehensive method that covers all enzymes in the genome of an organism [211, 212]. As human cell-based datasets, such as the human cell atlas, continue to develop, scRNA-seq's potential for aiding our understanding of the heterogeneity and metabolic diversity of TAMs is becoming increasingly significant [211, 212]. However, the analysis of recent articles on single-cell RNA sequencing has the limitation of being relatively dependent on transcriptome profiles, whereas various metabolite levels, metabolic enzyme activities, and environmental regulatory mechanisms, may coordinate to determine specific metabolic pathways in tumor immunity. To compensate, researchers are leveraging genome-scale metabolic models (GSMMs), such as Compass, an innovative FBA algorithm, that utilizes transcriptomic data to model metabolic states of individual cells, facilitating the study of TAMs metabolism and novel cancer treatment targets [207]. It also aids in discerning metabolic activity variations and predicts connections between phenotypes and diverse metabolic pathways or reactions [207]. Concurrently, future research could employ high-throughput multi-omics analyses coupled with big data models, to furnish more information on the metabolic and functional heterogeneity of TAMs.

In this review, we have explored the heterogeneity of TAMs in the TME during the single-cell omics era and highlighted how metabolic reprogramming of TAMs by the tumor microenvironment is a significant driver of TAM heterogeneity. We have also summarized recent studies on immunotherapies targeting TAM metabolism. Due to the intricate interplay between cytokines and metabolites in the TME, many aspects of TAMs' physiological responses to changes in metabolic levels remain unclear. Additionally, it is still uncertain how these responses vary in primary tumors versus metastatic niches. Furthermore, the current research on the role of tumor cell-released metabolites in regulating macrophage plasticity and polarization is limited to a few metabolites. Concurrently, the tumor microenvironment comprises multiple stromal cells, including cancer-associated fibroblasts (CAFs), necessitating an understanding of their metabolic interplay with TAMs to achieve a comprehensive insight into microenvironmental metabolism. Also, given the complexity of the metabolic characteristics within the tumor immune microenvironment and its systemic influence, there is currently a dearth of effective drugs specifically targeting TAM metabolism and future research needs to dedicate more efforts towards the identification of efficacious targets. Thus, we are only beginning to understand how TAM metabolic reprogramming affects the phenotypic and functional heterogeneity of macrophages, and future research in these areas may provide new therapeutic strategies for cancer treatment.

## Data Availability

Not applicable.

## Abbreviations

2-DG: 2-Deoxy-D-glucose
ACLY: ATP Citrate Lyase
ACOD1: Aconitate Decarboxylase
ADMA: Asymmetric Dimethylarginine
ADT: Androgen Deprivation Therapy
Ang2: Angiopoietin-2
ARRDC1: Arrestin Domain-Containing Protein 1
Arg1: Arginase-1
AHR: Arylhydrocarbon Receptor
BCKAs: Branched-Chain Ketoacids
bFGF: Basic fibroblast growth factor
BMDMs: Bone Marrow-Derived Macrophages
CAFs: Cancer-Associated Fibroblasts
CAR: Chimeric Antigen Receptor
CAT-1: Cationic Amino Acid Transporters
CPT1A: Carnitine Palmitoyltransferase 1A
COX2: Prostaglandin G/H Synthase 2
CRC: Colorectal Cancer
CRPC: Castration-Resistant Prostate Cancer
CSF1: Colony Stimulating Factor 1
CSF1R: Colony Stimulating Factor 1 Receptor
ER: Endoplasmic Reticulum
FABP: Fatty Acid Binding Protein
FAO: Fatty Acid Oxidation
FASN: Fatty Acid Synthase
FFAs: Free Fatty Acids
FH: Fumarate Hydratase
GPCRs: G-protein Coupled Receptors
GLUT-1: Glucose Transporter 1
GS: Glutamine Synthetase
GSMMs: Genome-Scale Metabolic Models
HA: Hyaluronic Acid
HCA1: Hydroxycarboxylic acid receptor 1
HCC: Hepatocellular Carcinoma
HGG: High-Grade Glioma
ICT: Immune Checkpoint Therapy
IDO: Indoleamine-2,3-Dioxygenase
ILC2s: Innate Lymphoid Cells Type 2
IRG1: Immune Response Gene 1 Protein
ITA: Itaconic Acid
KIC: α-Ketoisocaproic Acid
KMV: α-Keto-β-Methylpentanoic Acid
KIV: α-Ketoisovaleric Acid
LAMs: Lipid-Associated TAMs
LCFAs: Long-Chain Fatty Acids
LPL: Lipoprotein Lipase
LUAD: Lung Adenocarcinoma
MCT1: Monocarboxylate Transporter Protein 1
MCAD: Medium-Chain Acyl-CoA Dehydrogenase
MDA5: Melanoma Differentiation-associated Protein 5
MGLL: Monoacylglycerol Lipase
MMPs: Matrix Metalloproteinases
MSR1: Macrophage Scavenger Receptor 1
mtRNA: Mitochondrial RNA
NO: Nitric Oxide
NOS2: Nitric Oxide Synthase 2
NSCLC: Non-Small Cell Lung Cancer
OXPHOS: Oxidative Phosphorylation
PAC: Pancreatic Adenocarcinoma
PDAC: Pancreatic Ductal Adenocarcinoma
PFKFB3: 6-Phosphofructo-2-kinase/fructose-2,6-biphosphatase 3
PKM2: M2 isoform of Pyruvate Kinase
PPARγ: Peroxisome Proliferator-Activated Receptor γ
REDD1: Regulated in Development and DNA Damage Responses 1
RIPK3: Receptor-Interacting Protein Kinase 3
ROS: Reactive Oxygen Species
RLR: Retinoic acid-inducible gene I (RIG-I)-like receptors
RTKs: Receptor Tyrosine Kinases
RTMs: Resident Tissue Macrophages
scRNA-seq: Single-Cell RNA Sequencing
SLE: Systemic Lupus Erythematosus
SREBP1: Sterol Regulatory Element-Binding Protein 1
SUCNR1: Succinate Receptor 1
TAMs: Tumor-Associated Macrophages
TCA cycle: Tricarboxylic Acid Cycle
TDEs: Tumor-Derived Exosomes
TDO: Tryptophan 2,3-Dioxygenase
TGF-β: Transforming Growth Factor-beta
TME: Tumor Microenvironment
TSP-1: Thrombospondin-1
TSA: Trichostatin-A
VEGF: Vascular Endothelial Growth Factor
